# Stem Cell Pluripotency Genes Klf4 and Oct4 Regulate Complex SMC Phenotypic Changes Critical in Late-Stage Atherosclerotic Lesion Pathogenesis

**DOI:** 10.1161/CIRCULATIONAHA.120.046672

**Published:** 2020-07-17

**Authors:** Gabriel F. Alencar, Katherine M. Owsiany, Santosh Karnewar, Katyayani Sukhavasi, Giuseppe Mocci, Anh T. Nguyen, Corey M. Williams, Sohel Shamsuzzaman, Michal Mokry, Christopher A. Henderson, Ryan Haskins, Richard A. Baylis, Aloke V. Finn, Coleen A. McNamara, Eli R. Zunder, Vamsidhar Venkata, Gerard Pasterkamp, Johan Björkegren, Stefan Bekiranov, Gary K. Owens

**Affiliations:** 1 Robert M. Berne Cardiovascular Research Center (G.F.A., K.M.O, S.K., A.N., C.M.W., S.S., C.A.H., R.H., R.A.B., C.A.M., E.R.Z., G.K.O.), University of Virginia, Charlottesville.; 2 Department of Biochemistry and Molecular Genetics (G.F.A., K.M.O., C.A.H., R.A.B., S.B.), University of Virginia, Charlottesville.; 3 Department of Biomedical Engineering (C.M.W., E.R.Z.), University of Virginia, Charlottesville.; 4 School of Medicine, Division of Cardiovascular Medicine, Department of Medicine (C.A.M.), University of Virginia, Charlottesville.; 5 Department of Cardiac Surgery, Tartu University Hospital, Estonia (K.S.).; 6 Integrated Cardio Metabolic Centre, Department of Medicine, Karolinska Institutet, Karolinska Universitetssjukhuset, Huddinge, Sweden (G.M., V.V., J.B.).; 7 Laboratory of Clinical Chemistry and Hematology, Division Laboratories and Pharmacy (M.M., G.P.), University Medical Center Utrecht, University Utrecht, The Netherlands.; 8 Department of Cardiology (M.M.), University Medical Center Utrecht, University Utrecht, The Netherlands.; 9 CVPath Institute, Inc, Gaithersburg, MD (A.V.F.).; 10 Department of Genetics and Genomic Sciences (J.B.), Icahn School of Medicine at Mount Sinai, New York.; 11 Icahn Institute of Genomics and Multiscale Biology (J.B.), Icahn School of Medicine at Mount Sinai, New York.

**Keywords:** coronary disease, single nucleotide polymorphisms, smooth muscle cell, smooth muscle differentiation, smooth muscle progenitor cell

## Abstract

**Background:**

Rupture and erosion of advanced atherosclerotic lesions with a resultant myocardial infarction or stroke are the leading worldwide cause of death. However, we have a limited understanding of the identity, origin, and function of many cells that make up late-stage atherosclerotic lesions, as well as the mechanisms by which they control plaque stability.

**Methods:**

We conducted a comprehensive single-cell RNA sequencing of advanced human carotid endarterectomy samples and compared these with single-cell RNA sequencing from murine microdissected advanced atherosclerotic lesions with smooth muscle cell (SMC) and endothelial lineage tracing to survey all plaque cell types and rigorously determine their origin. We further used chromatin immunoprecipitation sequencing (ChIP-seq), bulk RNA sequencing, and an innovative dual lineage tracing mouse to understand the mechanism by which SMC phenotypic transitions affect lesion pathogenesis.

**Results:**

We provide evidence that SMC-specific Klf4- versus Oct4-knockout showed virtually opposite genomic signatures, and their putative target genes play an important role regulating SMC phenotypic changes. Single-cell RNA sequencing revealed remarkable similarity of transcriptomic clusters between mouse and human lesions and extensive plasticity of SMC- and endothelial cell-derived cells including 7 distinct clusters, most negative for traditional markers. In particular, SMC contributed to a Myh11^-^, Lgals3^+^ population with a chondrocyte-like gene signature that was markedly reduced with SMC-*Klf4* knockout. We observed that SMCs that activate Lgals3 compose up to two thirds of all SMC in lesions. However, initial activation of Lgals3 in these cells does not represent conversion to a terminally differentiated state, but rather represents transition of these cells to a unique stem cell marker gene–positive, extracellular matrix-remodeling, “pioneer” cell phenotype that is the first to invest within lesions and subsequently gives rise to at least 3 other SMC phenotypes within advanced lesions, including Klf4-dependent osteogenic phenotypes likely to contribute to plaque calcification and plaque destabilization.

**Conclusions:**

Taken together, these results provide evidence that SMC-derived cells within advanced mouse and human atherosclerotic lesions exhibit far greater phenotypic plasticity than generally believed, with Klf4 regulating transition to multiple phenotypes including Lgals3^+^ osteogenic cells likely to be detrimental for late-stage atherosclerosis plaque pathogenesis.

## Abstract 2

Supplemental Digital Content is available in the text.

**Clinical Perspective**

**What Is New?**

- Smooth muscle cells in late-stage atherosclerotic lesions undergo transition through Lgals3^+^ to several states, including inflammatory and Klf4-dependent osteogenic phenotypes.
- Transcription factors Klf4 and Oct4 bind putative target genes associated with genetic risk for coronary artery disease.

**What Are the Clinical Implications?**

- Smooth muscle phenotypic switching produces cells that can be beneficial or detrimental to lesion stability and may be an important mechanism controlling risk of unstable atherosclerotic plaque and myocardial infarction or stroke.

Despite decades of research, we have little understanding of the origin and function of many cells that make up late-stage atherosclerotic lesions, as well as the mechanisms by which they control plaque stability and risk for plaque rupture leading to myocardial infarction or stroke.^1,2^ Rigorous lineage tracing studies in mouse models,^3–5^ and in human lesions using epigenetic marker analyses,^6^ have shown that smooth muscle cells (SMCs) are far more abundant and play a much more complex role in lesion pathogenesis than predicted based on marker gene analyses. Shankman et al found that >80% of SMC in atherosclerotic plaque have lost expression of contractile markers like Acta2.^3^ Furthermore, up to 30% of these have activated expression of Lgals3/Mac2, and a smaller percentage express markers of stem cells or myofibroblasts like Sca1/Ly6a or the platelet-derived growth factor (PDGF)-β receptor. SMC-specific *Klf4* knockout (Myh11-Cre^ERT2^ eYFP apoE Klf4^Δ/Δ^, SMC^Klf4-KO^) resulted in lesions that were 50% smaller, exhibited evidence for increased plaque stability including a doubling in the Acta2^+^ fibrous cap, and had a >60% decrease in SMC-derived Lgals3^+^ cells.^3^ As such, Klf4-dependent changes in SMC phenotype and subsequent effects appear to exacerbate lesion pathogenesis. In contrast, SMC-specific *Oct4* knockout (Myh11-Cre^ERT2^ eYFP apoE Oct4^Δ/Δ^, SMC^Oct4-KO^) resulted in opposite effects including increases in lesion size and evidence for reduced plaque stability including the nearly complete absence of an SMC-enriched Acta2^+^ fibrous cap, reduced mature collagen content, increased lipid content, and increased intraplaque hemorrhage.^4^ Recent work by Wirka et al used single-cell (sc) RNA sequencing (RNA-seq) in combination with lineage tracing to define the transcriptional signature of SMC-derived cells in atherosclerosis, detecting an Lgals3^+^ cluster expressing genes for multiple ECM proteins.^7^ However, their analyses were performed on aortic root segments such that the majority of SMC and other cells analyzed were derived from the medial and adventitial layers, not lesions, thus severely limiting their sensitivity in detecting SMC lesion phenotypes. Moreover, their conclusion that SMCs give rise to a single so-called beneficial “fibrocyte” phenotype is incompatible with results of SMC-specific knockout studies clearly establishing that SMCs can play either a detrimental or beneficial role in plaque stability.^3,4^ As such, further definition of SMC subsets within lesions is critical, with the hope of identifying factors and mechanisms that promote beneficial SMC phenotypic transitions as novel therapeutic targets.

To better define the cellular origins and phenotypic properties of SMC and non-SMC within atherosclerotic lesions, we used a combination of bulk and scRNA-seq of advanced brachiocephalic artery (BCA) lesions from SMC-specific lineage tracing apoE^-/-^ mice with or without SMC specific conditional knockout of Klf4 or Oct4. Given the profound differences in lesion pathogenesis in these 2 knockout models, we hypothesized that studies would provide insights about not only the complexity of phenotypes exhibited by SMC, but also if these changes are likely to be beneficial or detrimental for late-stage plaque pathogenesis. Remarkably, we provide evidence that Klf4 and Oct4 control nearly opposite patterns of gene expression in SMC and based on in vivo ChIP-seq analyses have identified >80 potential Klf4 or Oct4 target genes that may impact SMC phenotypic transitions important in lesion pathogenesis. In addition, scRNA-seq studies on a unique dual recombinase lineage mouse generated by our laboratory and our previously published SMC-Klf4 knockout mice show that several SMC lesion phenotypes are derived from a subset of Lgals3^+^ transitional state SMCs that initially exhibit an extracellular matrix remodeling phenotype but ultimately contribute to multiple transcriptomic clusters, including populations of osteogenic and proinflammatory state cells likely to be detrimental for lesion pathogenesis.

## Methods

Data are available on request from the authors.

### Mice

All experiments followed guidelines of the University of Virginia Animal Care and Use Committee (Protocol 2500).

SMC^Klf4^ and SMC^Oct4^ mice were described previously.^3,4^ Littermate controls were used for all studies. Rosa-tdTomato-eGFP mice were obtained from Jackson Labs (stock No. 026931). Myh11-Dre^ERT2^ mice and Lgals3–internal ribsosomal entry site–Cre mice were made by Cyagen Labs. The *myosin heavy chain 11 (Myh11*) promoter was placed upstream of a Dre recombinase fused to an *estrogen receptor 2 (ERT2*) domain. Mouse embryos were injected with this transgene, which randomly inserted into the genome. For the Lgals3-Cre mice, a herpesvirus internal ribsosomal entry site followed by a Cre recombinase was integrated into the endogenous Lgals3 locus after the stop codon in exon 6. All mice were injected with 0.1 mL tamoxifen (1 mg/mL, Sigma No. T-5648) dissolved in peanut oil in 10 injections from 6 to 8 weeks of age. Mice were fed a Western diet containing 21% milk fat and 0.15% cholesterol (Tekland) for 18 weeks (10 weeks when noted). Because the Myh11-Cre^ERT2^ is located on the Y chromosome, only male mice were analyzed in these studies. However, because Myh11-Dre^ERT2^ does not have this limitation, both male and female mice were analyzed.

### Bulk RNA-seq

Total RNA was isolated using Trizol (Invitrogen) from the BCA and aortic arch region of SMC^-*Klf4*-WT/WT^, SMC^-*Klf4*-Δ/Δ^, SMC^-*Oct4*WT/WT^, and SMC^-*Oct4*Δ/Δ^ (n=4 or 5) mice fed a high-fat Western diet for 18 weeks. The RNA library was prepared according to the Illumina RNA-Seq library kit.

One hundred nucleotide paired-end reads were mapped to the mm10 reference genome using STAR software version 2.4^8^ against the mouse genome release M21 (GRCm38.p6) from Gencode. A table of gene counts was generated using FeatureCounts.^9^ We used the DESeq2^10^ Bioconductor R package to identify differentially expressed genes at a 5% false discovery rate (*P* value adjusted≤0.05) using the Benjamini-Hochberg procedure to adjust *P* values. Gene set enrichment analysis was performed using Ingenuity Pathway Analysis.^11^ Significantly enriched pathways were identified using a 5% false discovery rate cutoff, and their enrichment significance was quantified using −log_10_ of *P* value adjusted. Data are presented as pathways downregulated or upregulated in knockout mice compared with wild-type control mice.

### Klf4 and Oct4 ChIP-seq

Segments of the aorta from the arch to the aortic root and up to the carotid bifurcation isolated from 18-week Western diet–fed SMC^-*Klf4*-WT/WT^, SMC^-*Klf4*-Δ/Δ^, SMC^-*Oct4*WT/WT^, and SMC^-*Oct4*Δ/Δ^ (n=15 each group) mice were snap-frozen in liquid nitrogen and then processed for ChIP assays using the Klf4- or Oct4-specific antibody (Figure I in the Data Supplement) and sequenced using Illumina TruSeq Chip Library Kit. Sequencing reads were aligned to the mouse genome release M21 (GRCm38.p6) using the BOWTIE alignment tool.^12^ These aligned reads were then processed, involving removal of duplicate reads and format conversions using the SAMtools^13^ suite and converted into bam/bai format and loaded in the Integrative Genomics Viewer for visualization. The reads were also converted to BED format, and peaks were identified using MACS2^14^ callpeaks function against the mouse genome with a q value <0.05.

### Atherosclerotic Plaque scRNA-seq

For murine studies, individual atherosclerotic plaques from the BCA were removed from underlying media with forceps and deposited into 1% BSA in PBS plus 1 µg/mL Actinomycin D (Gibco, No. 11805017). Two thousand cells in each group were targeted in Chromium 10X genomics libraries, which, after barcoding, were pooled and sequenced on the Illumina NextSeq, 150 cycle high-output.

Human atherosclerotic plaques were obtained with in-formed consent from 14 male and 4 female patients undergoing a primary carotid endarterectomy in the Athero-Express Biobank Study (www.atheroexpress.nl) approved by the University Medical Center Utrecht Medical Ethical Committee. Viable cells were sorted 1 cell per well and immediately frozen at –80°C until further processing using the Sorting and Robot-Assisted Transcriptome Sequencing (SORT-seq) protocol.^15^ Cells were then pooled in 1 library, and the aqueous phase was separated from the oil phase, followed by in vitro transcription and library construction using the CEL-Seq2 protocol.^16^ Detailed cell isolation protocols for humans and mice are in the Methods in the Data Supplement.

Gene-barcode matrices were analyzed in R using Seurat v3.^17^ Cells were filtered for 200 to 5,000 reads per unique molecular identifiers, ≤10% mitochondrial and <5% hemoglobin gene content. Significant principal components of variation were calculated using JackStraw test with 10,000 repetitions, and clusters were calculated with 19 principal components of variation. Raw data are available at GSE150644. Differential expression analysis was done using Model-Based Analysis of Single-Cell Transcriptomics (MAST). Code is available on request.

### Immunofluorescent Staining and Quantification

Paraffin BCA sections were deparaffinized, rehydrated, antigen-retrieved (citrate), and blocked with fish skin gelatin PBS (6 g/L) containing 10% horse serum for 1 hour at room temperature. Frozen sections were permeabilized with acetone at –20° for 10 minutes. Slides were incubated with the antibodies shown in Table I in the Data Supplement. Slides were imaged using a Zeiss LSM700 confocal microscope to acquire a series of z-stack images at 1-μm intervals. Maximal intensity projection was used to generate the representative images included in the figures, and Fiji was used to process and format images.

### Statistical Analysis

Statistics were performed using GraphPad Prism 7. For comparison of 2 groups of continuous variables with normal distribution and equal variances, 2-tailed unpaired Student t tests (with additional Welch correction for unequal variances) were performed with a significance threshold of *P*≤0.05. For multiple group comparison, we performed 1-way or 2-way ANOVA followed by the Sidak method of multiple pairwise comparisons. The number of mice used for each analysis is indicated in the figure legends, selected by a power analysis to detect a 30% change with 10% error and 95% confidence.

## Results

Klf4 and Oct4 control opposite patterns of gene expression in late-stage atherosclerotic lesions and may regulate genes associated with human coronary artery disease (CAD) risk.

**Figure 1.**
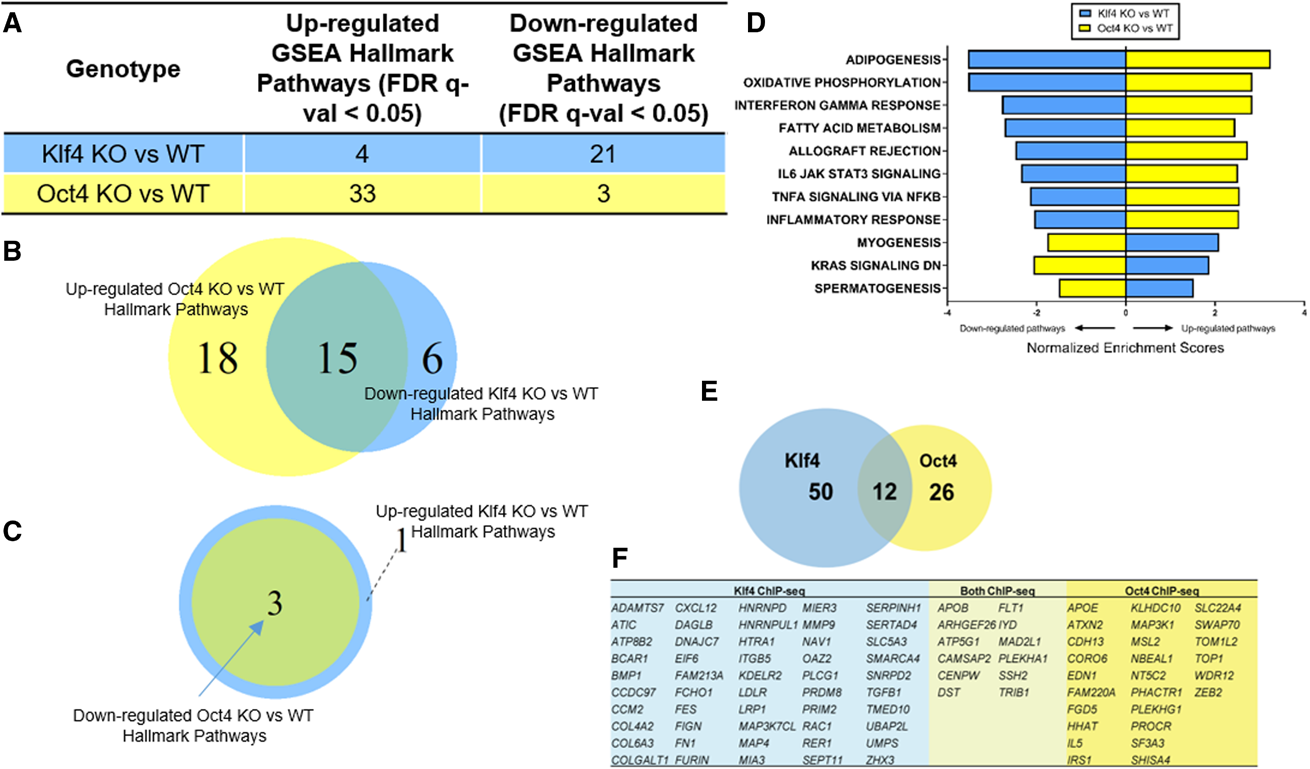
**Bulk RNA-seq and SMC^Oct4^ and SMC^Klf4^ ChIP-seq analysis of advanced BCA lesion areas from SMC-specific *Klf4* vs *Oct4* knockout apoE^-/-^ mice showed virtually opposite genomic signatures and identify candidate SMC Klf4 or Oct4 target genes that may play an important role in late-stage atherosclerotic lesion pathogenesis.** RNA-seq was performed on the BCA regions from 18-week Western diet–fed SMC^*Klf4*-Δ/Δ^ vs SMC^*Klf4*-WT/WT^ and SMC^*Oct4*-Δ/Δ^ vs SMC^*Oct4*-WT/WT^ mice. **A**, GSEA analysis showing MSigDB Hallmark pathways from comparing KO vs WT of both lines are shown. **B**, Venn diagram showing the common pathways between downregulated SMC^*Klf4*-Δ/Δ^ vs SMC^*Klf4*-WT/WT^ and upregulated in SMC^*Oct4*-Δ/Δ^ vs SMC^*Oct4*-WT/WT^. **C**, Venn diagram showing the common pathways between upregulated SMC^*Klf4*-Δ/Δ^ vs SMC^*Klf4*-WT/WT^ and downregulated in SMC^*Oct4*-Δ/Δ^ vs SMC^*Oct4*-WT/WT^. **D**, Bar graph showing the top 8 common pathways from **B** and all common pathways in **C** differentially regulated pathways between SMC^*Klf4*-Δ/Δ^ vs SMC^*Klf4*-WT/WT^ and SMC^*Oct4*-Δ/Δ^ vs SMC^*Oct4*-WT/WT^ analyses. **E**, ChIP-seq was done on the BCA regions from 18-week Western diet–fed SMC^*Klf4*-Δ/Δ^ vs SMC^*Klf4*-WT/WT^ and SMC^*Oct4*-Δ/Δ^ vs SMC^*Oct4*-WT/WT^ mice (n=15). The Venn diagram shows overlap of putative “closest gene” binding partners of Klf4 and Oct4 in SMC (ie, those binding partners that are lost in the SMC^*Klf4*-Δ/Δ^ compared with SMC^*Klf4*-WT/WT^) that are also human CAD GWAS loci. *P* value of gene overlap <0.01. **F**, Table showing genes present in the Venn diagram in **E**. BCA indicates brachiocephalic artery; CAD, coronary artery disease; ChIP-seq, chromatin immunoprecipitation sequencing; FDR, false discovery rate; GSEA, gene set enrichment analysis; GWAS, genome-wide association study; KO, knockout; RNA-seq, RNA sequencing; SMC, smooth muscle cell; and WT, wild-type.

To study global changes in gene expression that accompanied our atherosclerotic phenotypes of Klf4 and Oct4 knockout in SMCs, we conducted Klf4 and Oct4 ChIP-seq as well as bulk RNA-seq on the BCA lesion area of SMC^Klf4^ and SMC^Oct4^ knockout and wild-type mice after 18 weeks of Western diet (Figure I in the Data Supplement). By comparing ChIP-seq data between wild-type littermate control mice and our knockout mice, we were able to distinguish putative binding targets for each transcription factor in SMC, as well as any secondary changes in other cell types. Consistent with our previous data that SMC^Klf4^ and SMC^Oct4^ knockout have opposite phenotypes in terms of lesion morphology and cell composition, gene set enrichment analysis of bulk RNA-seq data using the MSigDb showed >70% of the down-regulated hallmark and Kyoto Encyclopedia of Genes and Genomes pathways in SMC^*Klf4*-Δ/Δ^ versus SMC^*Klf4*-WT/WT^ were up-regulated in SMC^*Oct4*-Δ/Δ^ versus SMC^*Oct4*-WT/WT^, and nearly all up-regulated pathways in SMC^*Klf4*-Δ/Δ^ versus SMC^*Klf4*-WT/WT^ were down-regulated in the SMC Oct4 knockout model (Figure 1A–1C, Figure II in the Data Supplement). Ingenuity Pathway Analysis of the ChIP-seq data showed that Oct4 putative targets in SMC were enriched for genes involved in pluripotency, cancer, and migration, whereas Klf4 putative targets in SMC were enriched for genes involved in leukocyte recruitment, actin regulation, and extracellular matrix organization (Figure IIIA–IIID in the Data Supplement, and example of ChIP-seq peaks, Figure III I and IIIJ in the Data Supplement). Of major interest, 88 genes linked with 39% (64/163) of accepted human CAD genome-wide association study (GWAS) loci^18^ were identified as the nearest gene binding target in SMC^Klf4^ or SMC^Oct4^ murine atherosclerotic lesions (Figure 1E and 1F, *P* value of gene overlap <0.01 and almost 2-fold [1.94 fold] higher than expected by chance). Extensive additional studies would be required to determine if Klf4 or Oct4 directly regulate or modulate any of these candidate GWAS loci. However, we have shown that approximately one third of these genes are regulated by Klf4 or Oct4 using an in vitro model of cholesterol loading (SMC^Klf4^) or hypoxia plus POVPC (1-palmitoyl-2-(5-oxo-valeryl)-sn-glycero-3-phosphorylcholine) (SMC^Oct4^, Figure IIIE and IIIF in the Data Supplement).

To further investigate the importance of *Klf4* and *Oct4* in human disease, we performed network analysis on the human STARNET database and identified a *Klf4* module of the atherosclerotic wall (Figure IV in the Data Supplement). This module was strongly associated with CAD (preoperative angiographically assessed DUKE score) and plasma low-density lipoprotein levels. In addition, genes present in this network module are upregulated in patients with CAD compared with CAD negative controls. Taken together, these results suggest that *Klf4* is not only a GWAS CAD gene^19^ but might also be regulating several previously reported CAD-associated gene loci.

### scRNA-seq Analysis of Advanced BCA Lesions Identified 14 Distinct Cell Clusters Including 7 of SMC Origin

**Figure 2.**
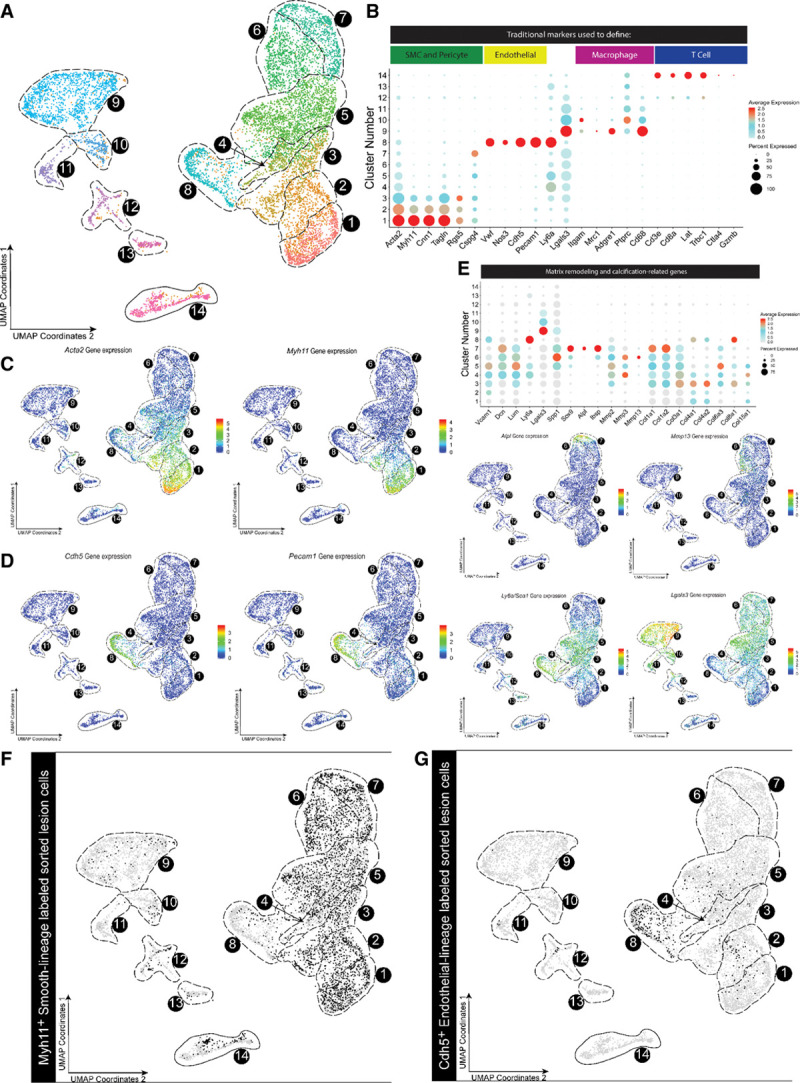
**scRNA-seq analysis of healthy aorta and advanced BCA lesions identified 14 distinct cell clusters including 7 predominately of SMC origin. A**, Cells were harvested for scRNA-seq from microdissected advanced BCA lesions from SMC lineage-traced (Myh11-Cre^ERT2^ Rosa-eYFP apoE^-/-^) or endothelial lineage-traced (Cdh5-Cre^ERT2^ Rosa-eYFP apoE^-/-^) 18-week Western diet–fed mice, as well as healthy 8-week-old apoE^-/-^ SMC lineage-traced mouse aorta as a control. In aggregate, these data are the results of 7 experiments, 19 Chromium 10X libraries, and 23 mice. Results are shown as a UMAP colored by cluster. **B**, Dot plot of traditional markers used for cell identity. **C**and **D**, Feature plots showing gene expression of Myh11, Acta2 (**C**) and Cdh5 and Pecam1 (**D**), as representative genes of classical markers for SMC and endothelial cells, respectively. *Myh11* was detected only in clusters 1 to 2, whereas *Cdh5* was detected exclusively in cluster 8. **E**, Dot plots and feature plots of selected genes representing 3 SMC phenotypic groups, stem/inflammatory, ECM remodeling, and osteogenic. Further plots and pathway analysis justifying the groups can be found in Figures V and VI in the Data Supplement. **F**and**G**, UMAP of mouse cells showing (**F**) Myh11-Cre^ERT2^ Rosa-eYFP apoE^-/-^ (black dots) or (**G**) Cdh5-Cre^ERT2^ Rosa-eYFP apoE^-/-^lineage-traced cells from BCA plaques of mice fed a Western diet for 18 weeks (black dots). BCA indicates brachiocephalic artery; scRNA-seq, single-cell RNA sequencing; SMC, smooth muscle cell; and UMAP, uniform manifold approximation and projection.

To better understand the heterogeneity of phenotypes that SMC can acquire within atherosclerotic lesions, we performed scRNA-seq analyses on advanced BCA lesions of our Myh11-Cre^ERT2^ Rosa-eYFP apoE^-/-^ SMC lineage tracing mice and Cdh5-Cre^ERT2^ Rosa-eYFP apoE^-/-^ endothelial cell lineage tracing mice fed a Western diet for 18 weeks. Contrary to previous scRNA-seq analysis in the field,^20–22^ instead of analyzing whole atherosclerotic aortas, we isolated late-stage lesions microdissected from the BCA. To efficiently detect transcriptome changes in lineage-traced SMC or EC and to avoid any issues with read depth on eYFP (enhanced yellow fluorescent protein) transcript detection, we also sorted lineage-traced eYFP^+^ cells from these late-stage lesions. We also analyzed a healthy aortic sample from chow-fed 8-week-old SMC lineage tracing apoE^-/-^ mice directly after inducing SMC lineage tracing to provide a baseline scRNA-seq profile of medial (nonlesion) cells. Clustered together, results showed 14 different uniform manifold approximation and projection transcriptomic clusters including clusters corresponding to macrophages, T cells, SMCs, and endothelial cells identified by traditional markers (Figure 2A–2C). Using data from Myh11-lineage traced sorted cells, we observed that SMCs were the major source of at least 7 different clusters (Figure 2F). Interestingly, most of the clusters containing lineage-traced SMCs do not express *Myh11* (Figure 2C) or other SMC contractile protein markers, but instead express markers such as *Vcam1*, *Dcn/Lum*, *Lgals3*, *Spp1*, and *Sox9* (Figure V in the Data Supplement). Using Reactome and gene ontology pathway analysis of the top 100 genes in each cluster (Figure VI in the Data Supplement, Excel File I in the Data Supplement), we broadly characterized these clusters as inflammatory (clusters 4 to 5, “immunoregulatory interactions”), ECM-rich (cluster 6, “extracellular matrix organization” and “degradation of extracellular matrix”), and osteogenic (cluster 7, “chondrocyte differentiation” and “bone mineralization”).

Similarly, we observed endothelial cells expressing SMC marker genes in clusters 1 to 3 that are likely related to endothelial-mesenchymal transition, including activation of *Acta2* and *Vimentin*. Interestingly, there were also EC-derived cells in clusters 2 to 5, consistent with phenotypes related to phenotypically modified SMC (Figure 2B and 2G, Figure VII in the Data Supplement). We did observe some SMC-derived cells in clusters defined by canonical macrophage markers; however, it has been reported that single-cell methodologies undersample macrophages in general by up to 65%, which may explain the low abundance of these cells.^20^ We also observed some SMC-derived cells in the T cell island (cluster 14), consistent with studies by Hansson et al^23^ showing that SMCs can take on antigen presentation properties within lesions. Taken together, these results suggest that cells from multiple origins exhibit extraordinary plasticity during lesion development, with cells acquiring alternative phenotypes to adapt to the extreme lesion environment.

### scRNA-seq Analysis of Advanced BCA Lesions From SMC^*Klf4*-*Δ*/Δ^ Versus SMC^*Klf*4-WT/WT^ apoE^-/-^ Mice Identified New Klf4-Dependent SMC Phenotypes

**Figure 3.**
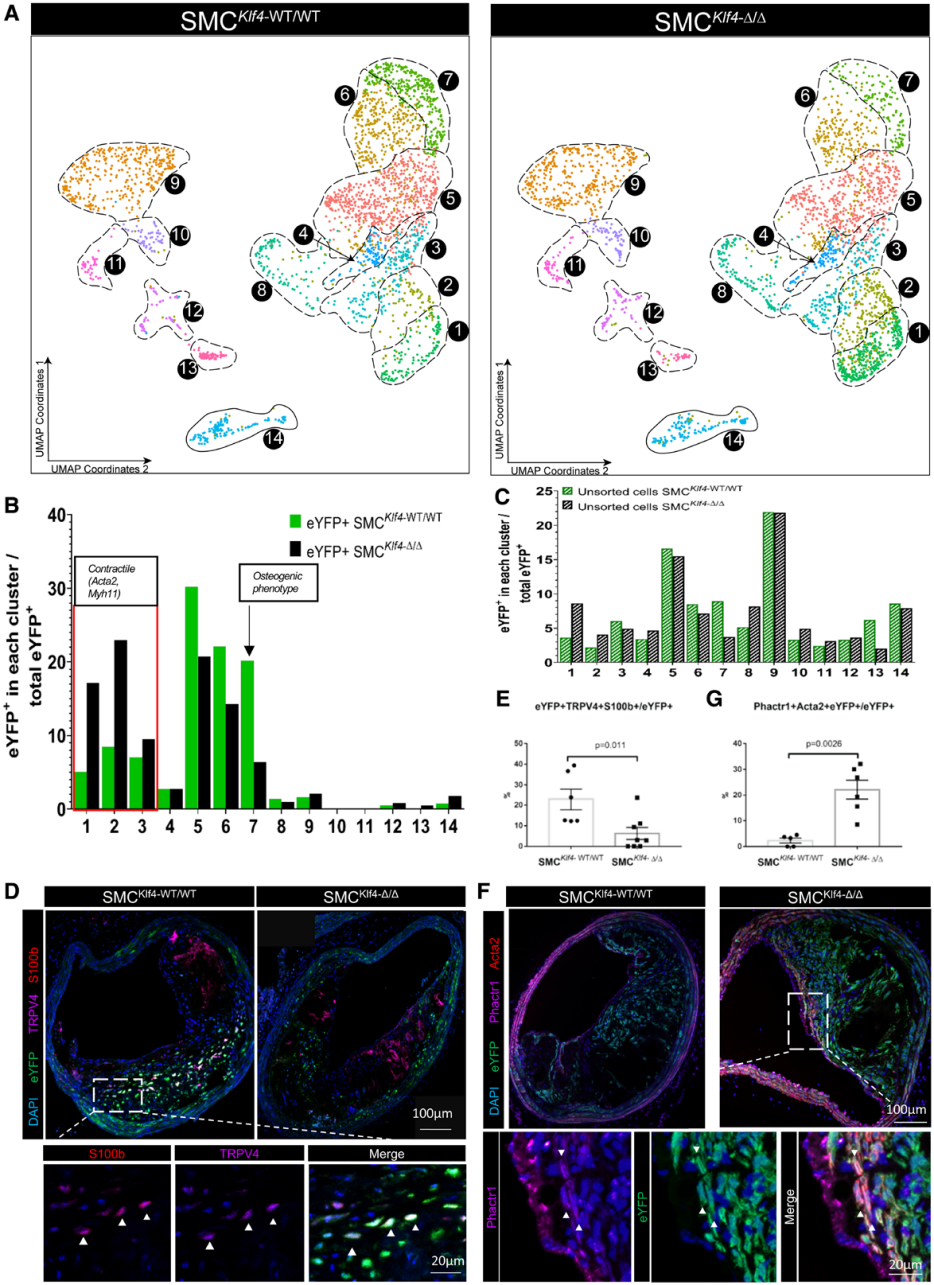
**scRNA-seq of advanced BCA lesions from SMC-specific *Klf4* knockout vs wildtype littermate control mice identified multiple Klf4-dependent clusters. A**, Cells from advanced BCA lesions were isolated from SMC^*Klf4*-WT/WT^ and SMC^*Klf4*-Δ/Δ^ mice after 18 weeks of Western diet and submitted for scRNA-seq. UMAP analysis shows cells from SMC^*Klf4*-WT/WT^ on the left and from SMC^*Klf4*-Δ/Δ^ cells on the right. The analysis includes 5469 cells from 2 different experiments (Chromium 10x runs) and 11 animals. **B**, Percentage of SMC-lineage traced cells (eYFP^+^ cells sorted from BCA plaque) in each UMAP cluster. **C**, Percentage of non-SMC lineage-tracing cells (unsorted cells) in each UMAP cluster from each respective group described in **A. D–G**, BCA lesions from SMC^*Klf4*-WT/WT^ and SMC^*Klf4*-Δ/Δ^ mice were stained for selected immunofluorescent markers from scRNA-seq clusters. **D**, Costaining for TRPV4 and S100b (markers present in cluster 7) with eYFP in SMC^*Klf4*-WT/WT^ and SMC^*Klf4*-Δ/Δ^ animals. Images are based on confocal microscopy z-stacks of 1 µm thickness on 10-µm sections of BCA lesions. The top left panel shows a maximum intensity projection ×20 zoom. A close-up view of eYFP^+^TRPV4^+^S100b^+^ cells is shown in a 1-µm individual z-stack in a WT animal. **E**, Quantification of the frequency of triple-positive (eYFP^+^ TRPV4^+^ S100B^+^) cells as a percent of total eYFP^+^ SMC-derived cells in SMC^*Klf4*-WT/WT^ and SMC^*Klf4*-Δ/Δ^ (n=6–8, error bars show SEM). **F**, Results of costaining for eYFP and Phactr1 (a marker of cluster 3) in advanced BCA lesions from SMC^*Klf4*-WT/WT^ and SMC^*Klf4*-Δ/Δ^ animals. The top panels show ×20 images. The bottom panels show a close-up image of a BCA lesion from SMC^*Klf4*-Δ/Δ^ animal. **G**, Quantification of the frequency of double-positive (eYFP^+^ Phactr1^+^) cells as a percent of total eYFP^+^ SMCs in advanced BCA lesions from SMC^*Klf4*-WT/WT^ and SMC^*Klf4*-Δ/Δ^ mice (n=6, error bars show SEM). BCA indicates brachiocephalic artery; scRNA-seq, single-cell RNA sequencing; SMC, smooth muscle cell; UMAP, uniform manifold approximation and projection; and WT, wild-type.

To determine mechanisms by which SMC conditional knockout of *Klf4* induced marked reductions in lesion size and increased indices of plaque stability, we performed scRNA-seq analyses on advanced BCA lesions from our SMC*Klf4*^-Δ/Δ^ versus SMC^*Klf*4-WT/WT^ apoE^-/-^ mice in the same manner as in Figure 2. Results showed that advanced BCA lesions from SMC*Klf4*^-Δ/Δ^ mice had a near doubling in the proportion of SMC-derived lesion cells expressing traditional markers in clusters 1 and 2 (Figure 3A and 3B). Furthermore, SMC^*Klf4*^ knockout resulted in a 4-fold decrease in the proportion of SMC-derived cells transitioning to an Lgals3^+^ osteogenic phenotype in cluster 7 (Figure 3A and 3B), characterized by expression of *Sox9, Runx2, Cytl1*, *Ibsp*,^24^ and *Alpl*, which is a key enzyme driving an osteogenic response in cells.^25–27^ In addition, differential gene expression analysis showed increased expression of *Acta2*, *Myh11*, and other SMC contractile markers, and a decrease in expression of *Spp1*, *Lgals3*, and *Sox9* in the SMC*Klf4*^-Δ/Δ^ versus SMC^*Klf4*-WT/WT^ advanced BCA lesions (Figure VIII in the Data Supplement, Excel File II in the Data Supplement with CAD loci–associated genes highlighted). There were also changes in non-SMC populations with SMC^*Klf4*^ knockout, including a 3-fold increase in cluster 13, marked by histocompatibility genes associated with activation of B cells and T cells.

To determine if transcriptomic clusters observed by scRNA-seq can also be identified at the protein level, we performed mass cytometry (CyTOF) analyses on cells isolated from advanced BCA lesions from SMC^*Klf4*-Δ/Δ^ versus SMC^*Klf4*-WT/WT^ mice using an antibody panel of 20 conventional markers supplemented with 11 novel markers identified in our scRNA-seq analyses (Figure IX and Table II in the Data Supplement). Further, to determine the location of these clusters within intact lesions, we stained sections of advanced mouse BCA lesions with antibodies to TRPV4, S100b, Sox9 (cluster 7), and Phactr1(cluster 2). High-resolution confocal microscopy identified eYFP^+^Trpv4^+^S100b^+^ cells (Figure 3D and 3E) in the body of the lesions whose frequency was significantly decreased by >50% in SMC^*Klf4*-Δ/Δ^ versus SMC^*Klf4*-WT/WT^. In contrast, eYFP^+^Acta2^+^Phactr1^+^ cells (Figure 3F and 3G) were increased 10-fold in SMC^*Klf4*-Δ/Δ^ versus SMC^*Klf4*-WT/WT^.

Taken together, our results indicate that loss of Klf4 in SMC is associated with marked decreases in lesion SMCs that exhibit an Lgals3^+^ osteogenic phenotype, which is of major interest given our previous studies in SMC Klf4 knockout mice that show loss of Lgals3-expressing SMC as well as decreased lesion size and increased fibrous cap thickness.^3^ Together with our finding that SMC^Klf4^ may regulate genes involved in genetic risk for CAD, this implies that Lgal3^+^ SMCs have negative implications for atherosclerosis pathogenesis. In addition, our scRNA-seq data indicate that *Lgals3* activation is not a specific marker of transition of SMC to a macrophage-like state but rather is first observed in transition clusters (Figure 2E) along with *Ly6a/Sca1* and *Vcam1*. Further, knockdown of Lgals3 in cultured SMCs prevented PDGF-induced expression of Sca1 (Figure X in the Data Supplement), suggesting *Lgals3* marks a key transitional state.

### Studies With a Novel SMC^-Dual Lineage^ Tracing Mouse Show That >60% of Lesion SMCs Activate *Lgals3* and Give Rise to Multiple Downstream Nonmacrophage SMC Clusters

**Figure 4.**
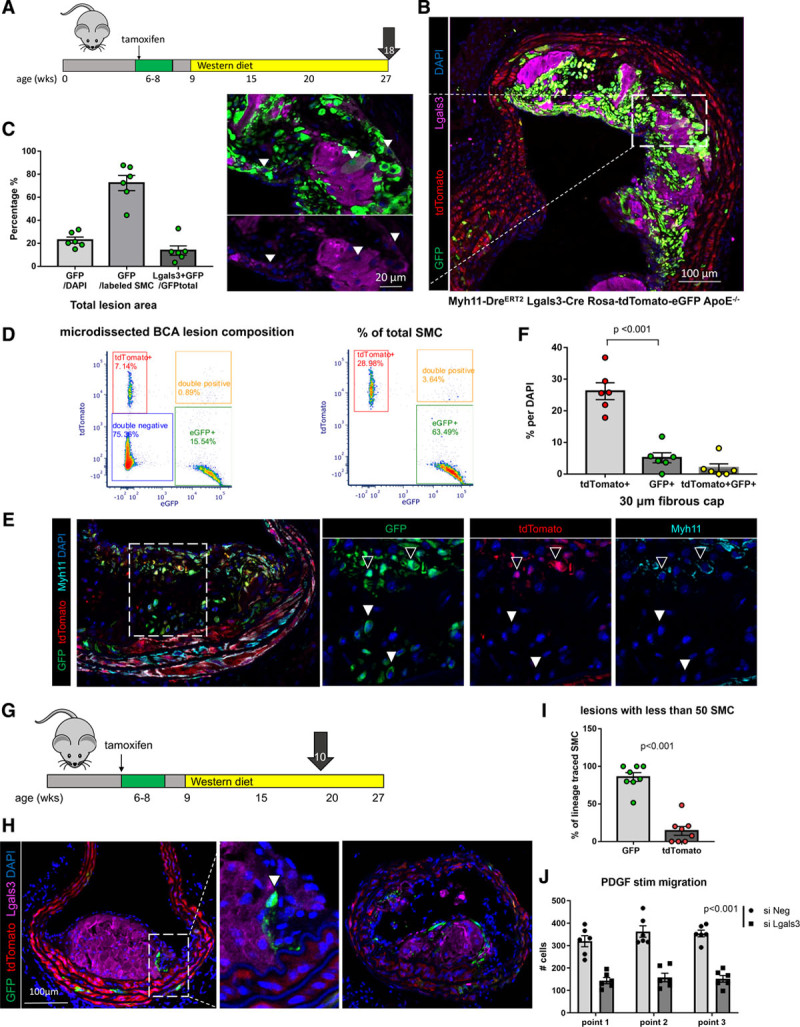
**A large fraction of Myh11-tdTomato^+^ SMCs phenotypically transition through an Lgals3 activation step and accumulate within advanced BCA lesions of Myh11-Dre^ERT2^ Lgals3-Cre Rosa-tdTomato-eGFP apoE^-/-^ mice. A**, Experimental design; SMC^Dual Lineage^ (Myh11-Dre^ERT2^ Lgals3-Cre Rosa-tdTomato-eGFP apoE^-/-^) mice were injected with tamoxifen at 6 to 8 weeks of age and subsequently placed on Western diet for 18 weeks to induce advanced atherosclerosis. Video of mouse model is available at https://www.cvrc.virginia.edu/Owens/educationaltopics.html. B, Sections of the BCA at the origin from the aorta in an SMC^Dual Lineage^ mouse, immunostained for GFP, tdTomato, and Lgals3, were imaged with confocal microscopy z-stacks of 1 µm thickness. Picture at right shows the maximum-intensity projection image of the 10-µm tissue thickness, and the close-up view shows eGFP^+^ Lgals3^+^ cells within a 1-µm individual z-stack. **C**, Quantification of cells positive for eGFP or Lgals3 by single-cell counting in 1-µm z-stacks of advanced BCA lesions from Myh11-Dre^ERT2^ Lgals3-Cre Rosa-tdTomato-eGFP apoE ^-/-^ mice (n=6, error bars show SEM). **D**, Flow-cytometric evaluation of cells freshly isolated from microdissected BCA lesions from Myh11-Dre^ERT2^ Lgals3-Cre Rosa-tdTomato-eGFP apoE^-/-^ BCA mice (n=4). These analyses included gating on live, single cells with fluorescence-minus-1 controls. Results show clear resolution of tdTomato^+^, eGFP^+^, and dual tdTomato^+^ eGFP^+^ (transition state) cells with normalization to the total number of lesion cells in the left panel or lineage-traced SMC in the right panel. **E**, Immunostaining of BCA lesions from Myh11-Dre^ERT2^ Lgals3-Cre Rosa-tdTomato-eGFP apoE^-/-^ 18-week Western diet–fed mice for GFP, tdTomato, and Myh11 reveals rare tdTomato^+^GFP^+^ cells in the fibrous cap (open arrows), overlying GFP^+^-only cells (filled arrows). **F**, Quantification of the proportion of cells in the fibrous cap of advanced BCA lesions that were tdTomato^+^, GFP^+^, or double-positive as a percent of total DAPI-stained fibrous cap cells. The fibrous cap was defined as the 30-µm lesion area underlying the lumen (n=6, error bars show SEM; the *P* value refers to unpaired *t* test between tdTomato^+^ and GFP^+^ cells). **G**, Experimental design; Myh11-Dre^ERT2^ Lgals3-Cre Rosa-tdTomato-eGFP apoE^-/-^ mice were injected with tamoxifen between 6 and 8 weeks of age and put on Western diet for 10 weeks to induce early atherosclerotic lesions. Ten animals were sampled at 4 locations in the BCA and imaged to capture early-stage lesions with <50 SMCs based on counting of lineage-traced SMC-derived cells in the entire lesion. **H**, Immunostaining for GFP, tdTomato, and Lgals3 by confocal microscopy as in **A. I**, Quantification of GFP^+^ and tdTomato^+^ cells in lesions of <50 lineage-traced SMCs total (n=8, error bars show SEM, *P*<0.01 by unpaired *t* test), **J**, SMCs isolated from Myh11 lineage tracing mice were treated with siLgals3 or a scrambled control siNeg, serum starved, and scratched with a pipet tip in 10% serum media + 10 µg/mL PDGF-BB. Light microscopic images of 3 points along the scratch were each taken at 0, 24, and 48 hours, and cells within the scratch area defined at time 0 hours were quantified (n=6, error bars show SEM). BCA indicates brachiocephalic artery; GFP, green fluorescent protein; PDGF, platelet-derived growth factor; siLgals3, small interfering RNA to Lgals3; and SMC, smooth muscle cell.

**Figure 5.**
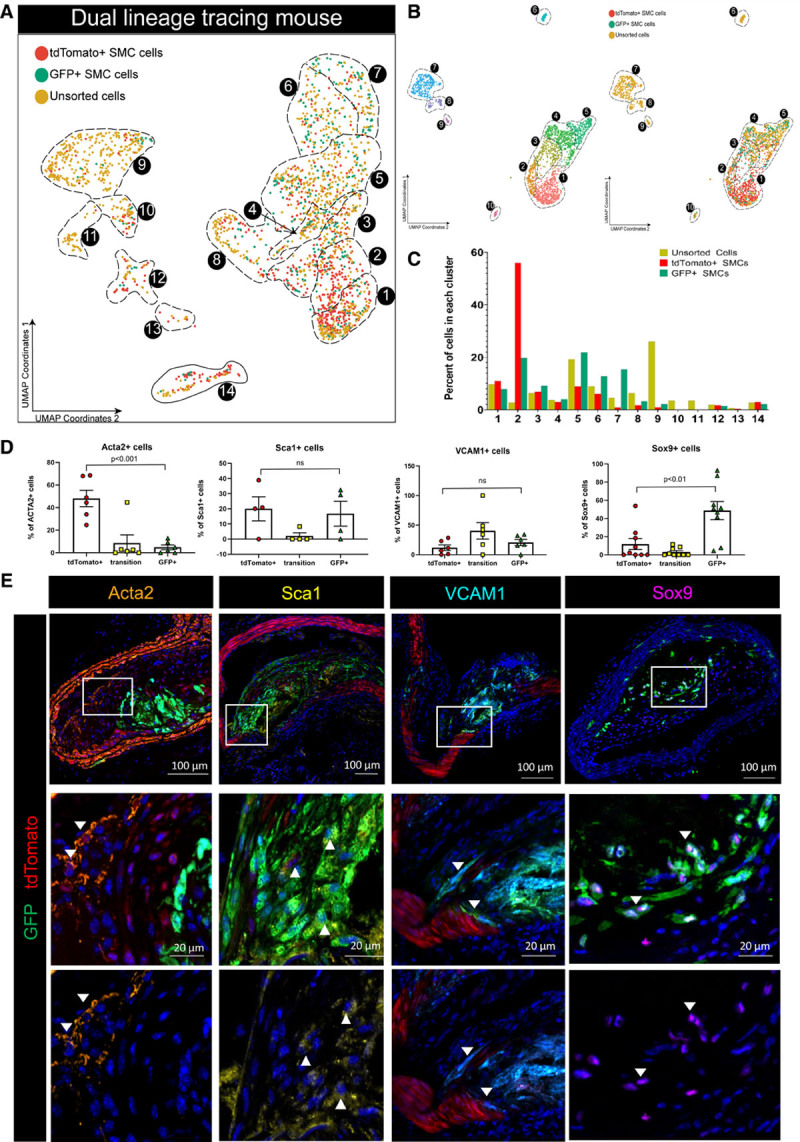
**Myh11-expressing differentiated SMC that transition through an Lgals3 activation state take on multiple phenotypes. A**, scRNA-seq was performed on BCA plaque cells isolated from Myh11-Dre^ERT2^ Lgals3-Cre Rosa-tdTomato-eGFP apoE^-/-^ mice fed a Western diet for 18 weeks, as well as eGFP^+^ and tdTomato^+^ cells sorted from plaque or whole atherosclerotic aorta. The results of UMAP analyses are shown colored by the library of origin. Results are from 2143 cells from 3 separate Chromium 10x runs and 6 animals. **B**, UMAP of analysis based on only cells from Myh11-Dre^ERT2^ Lgals3-Cre Rosa-tdTomato-eGFP apoE^-/-^ experiments showing clear separation of tdTomato^+^ and GFP^+^ (right side). **C**, Quantification of unsorted, GFP^+^, and tdTomato^+^ cells in each cluster in **A. D** and **E**, Atherosclerotic lesions from Myh11-Dre^ERT2^ Lgals3-Cre Rosa-tdTomato-eGFP apoE^-/-^ were immunostained for major markers of GFP^+^ clusters from our scRNA-seq analyses and Acta2. Frozen sections (10 µm) of renal arteries were used for VCAM1 and Sca1 antibody staining, along with tdTomato signal (endogenous) and a GFP antibody. Paraffin sections (10 µm) of BCA were used for Acta2 and Sox9. Because of limited available antibodies, Sox9 required staining for tdTomato and GFP on sequential sections, which were used for quantification in **D**. Sox9 and GFP are shown as the representative image in **E**. Sox9 positivity was not found on SMCs outside the most advanced BCA lesions, including renal and aortic root sections. BCA indicates brachiocephalic artery; GFP, green fluorescent protein; scRNA-seq, single-cell RNA sequencing; SMC, smooth muscle cell; and UMAP, uniform manifold approximation and projection.

To rigorously define the phenotype and potential functional properties of SMC-derived Lgals3^+^ lesion cells, we generated a novel dual lineage tracing model that uses sequential activation of Dre and Cre recombinases to track the transitions of SMC from Myh11^+^ to Lgals3^+^ states in adult mice. In brief, the Myh11 promoter drives a tamoxifen-inducible Dre recombinase, which removes a roxed stop cassette in the *Rosa* locus in front of a tdTomato-STOP-eGFP (enhanced green fluorescent protein) fluorescent reporter and a stop cassette on the *Lgals3* locus in front of an internal ribsosomal entry site–Cre. If this cell later goes on to express *Lgals3*, it will express Cre and remove the *tdTomato* flanked by loxP sites, allowing expression of eGFP. Figures XI and XII in the Data Supplement provide an overview and extensive validation of this mouse model. At baseline, medial SMCs of the aorta are tdTomato^+^ only. However, after 18 weeks of Western diet, eGFP^+^ cells accumulate in atherosclerotic plaques of the BCA, aortic arch, and renal arteries (Figures 4A, 4B, and 5E). These eGFP^+^ cells make up 60% to 80% of all advanced lesion SMCs measured by both flow cytometry and high-resolution confocal microscopy (Figure 4C and 4D). Original SMC lineage tracing placed the number of Lgals3^+^ SMC-derived cells at 30%^3^; however, because our dual lineage tracing system is cumulative, SMCs that expressed Lgals3 at any time will remain eGFP^+^. Indeed, only 25% of eGFP^+^ SMCs in lesions continued to show surface Lgals3 immunostaining (Figure 4C).

To test the hypothesis that SMCs transitioned through Lgals3 take on multiple phenotypes in lesions, we performed scRNA-seq analyses on advanced BCA plaques from our Myh11-Dre^ERT2^ Lgals3-Cre Rosa-tdTomato-eGFP apoE^-/-^ (SMC^Dual Lineage^) mice fed a Western diet for 18 weeks, along with GFP^+^ cells sorted from lesions and tdTomato^+^ sorted from whole aortas with plaque to identify clusters containing SMC-derived cells (Figure 5A). Consistent with Lgals3 expression in Figure 2, the lower clusters contain tdTomato^+^ cells expressing multiple contractile SMC markers, whereas more than half of all eGFP^+^ cells localize in clusters 5 to 7 expressing *Lgals3*, inflammatory genes (*Ccl2, Vcam1)*, ECM genes (*Dcn, Lum, Mmp3*), or *Sox9*, *Runx2*, and *Spp1*, the signature of the osteogenic phenotype cells decreased with SMC^Klf4^ knockout (Figures 3B and 5C, Figure V in the Data Supplement). We further validated the existence of these cells using high-resolution confocal analysis, showing that tdTomato^+^ cells dominate Acta2^+^ SMCs but GFP^+^ cells dominate Sox9^+^ SMCs (Figure 5D and 5E). Taken together, our results suggest that instead of a terminal marker of SMC switched to a macrophage-like state as originally postulated,^3,5^ *Lgals3* activation in these cells appears to be a marker of an earlier transitional state from which cells may subsequently exhibit multiple different phenotypes.

### SMCs Transitioned Through *Lgals3* Dominate Initial Investment of SMCs Into Lesions

In our early analyses of SMC^Dual Lineage^ mice, there were a small number of tdTomato^+^eGFP^+^ double-positive cells observed by flow cytometry (2%) and confocal microscopy within plaques (Figure 4D and 4E). Given that the half-life of tdTomato is 3 and a half days,^28^ we reasoned that these cells represented SMC currently in the process of transition (ie, cells have excised the *tdTomato* sequence and activated eGFP but not yet fully degraded the tdTomato protein). We also were able to observe tdTomato^+^eGFP^+^ cells in explants of aortas that lose tdTomato^+^ status over time (Figure XIII in the Data Supplement, Movies I and II in the Data Supplement) and detected double-positive cells by CyTOF (Figure XIV in the Data Supplement). We also made sure that tdTomato^+^eGFP^+^ cells did not represent cells that were poorly recombined at only 1 Rosa locus by observing this phenomenon in animals with only 1 Rosa-tdTomato-eGFP allele (Figure XV in the Data Supplement). Using confocal microscopy, we observed tdTomato^+^eGFP^+^ transitional SMC in the fibrous cap (Figure 4E) as well as the underlying media, often expressing VCAM1 (Figure 5E). Because these cells exist at the time of harvest after 18 weeks of Western diet, their double-positive status suggests that the transition of Myh11^+^ cells to a Lgals3^+^ state is an ongoing process throughout plaque pathogenesis.

Because this transition happened in the late-stage cap, we postulated that early-stage lesions would be dominated by tdTomato^+^ SMC. We fed SMC^Dual Lineage^ mice a Western diet for 10 weeks and examined lesions at the beginnings of SMC investment, defined as <50 lineage-traced SMC per plaque. Contrary to our expectations, we observed that almost all the SMC involved in early-stage lesions were eGFP^+^ (Figure 4G through 4I). These eGFP^+^ cells surrounded non-SMC–derived macrophage foam cells, consistent with a recent report suggesting that SMCs first populate lesions via the fibrous cap.^29^ However, the mature fibrous cap is dominated by tdTomato^+^ cells (Figure 4E and 4F) and features occasional transition cells directly overlying cells that were GFP^+^ only. Of note, Sox9^+^ GFP^+^ SMCs were observed in advanced but not early-stage lesions. Even if eGFP^+^ “pioneers” could revert to a Myh11^+^Acta2^+^Lgals3^-^ state, they cannot return to being tdTomato^+^. Thus, our data suggest that eGFP^+^Lgals3^+^ cells have privileged capacity to migrate into early-stage lesions but are later overtaken by tdTomato^+^ cells that form the stable fibrous cap (Figure XVI in the Data Supplement). Although we originally selected Lgals3 purely as a marker of phenotypically modulated SMC, in vitro studies suggest Lgals3 may have a functional role, with small interfering RNA knockdown of Lgals3 reducing SMC migration and transcriptional activation of several collagens in response to PDGF-BB (Figure 4J, Figures X and XVII in the Data Supplement).

### scRNA-seq and Confocal Analysis of Human Atherosclerotic Lesions Show Similar Transcriptomic Cell Subsets Between Human and Mouse Lesions

**Figure 6.**
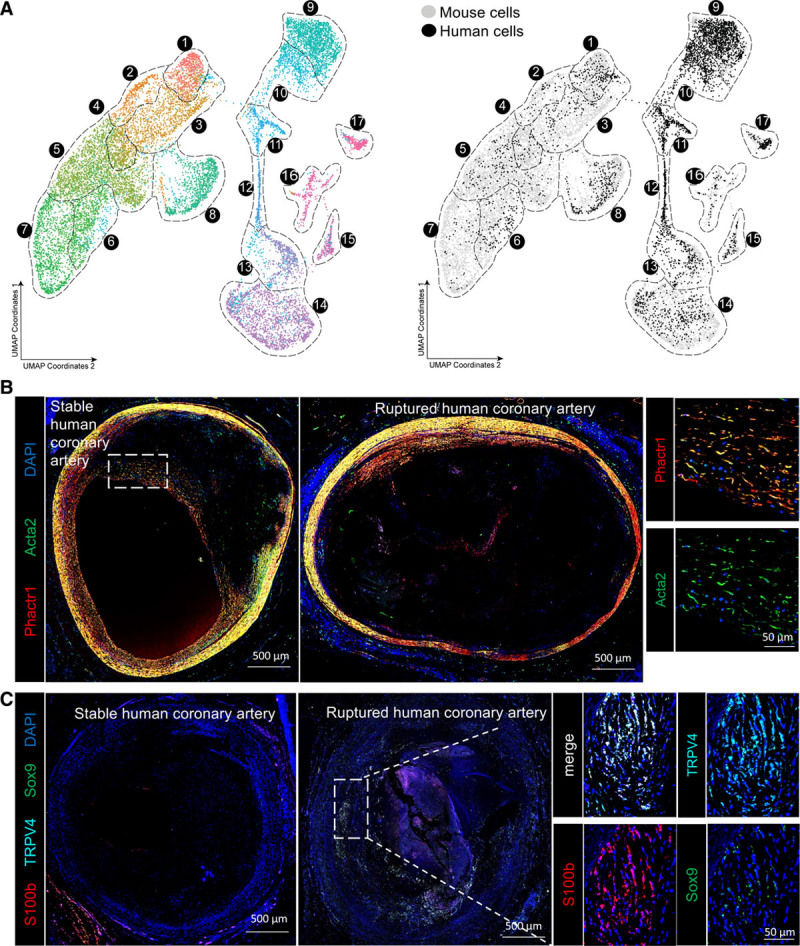
**Human scRNA-seq shows similar groups of fibrous cap and osteogenic SMC in advanced lesions. A**, UMAP analysis of scRNA-seq results from human carotid endarterectomy samples, colored by cluster (left) or by origin (right) against mouse scRNA-seq data from Figure 2. **B**, Human coronary artery lesions graded as stable or ruptured were immunostained for Acta2 and Phactr1, a marker of cluster 1 to 3 SMCs identified in our scRNA-seq analyses of advanced BCA lesions. Images of whole immunostained human coronary lesions were obtained by taking a tile scan of ×20 zoom confocal microscopy z-stacks of full 10-µm thickness. **C**, Human coronary artery lesions graded as stable or ruptured were immunostained for Sox9, TRPV4, and S100b, which were markers of osteogenic SMCs identified in our scRNA-seq analyses of mouse advanced BCA lesions, imaged as described in **B**. BCA indicates brachiocephalic artery; scRNA-seq, single-cell RNA sequencing; SMC, smooth muscle cell; and UMAP, uniform manifold approximation and projection.

One of the strengths of scRNA-seq analysis is the ability to compare cell types resolved using different technologies and even species.^17^ Therefore, we tested if the populations observed in our mouse models were present in human lesions via scRNA-seq. Of major significance, all clusters present in mouse advanced lesions were present in the human lesions, including cells in the osteogenic phenotype that was reduced with SMC^Klf4^ knockout (Figure 6A, Figure XVIII in the Data Supplement). In addition, we validated these findings using confocal microscopy showing that specific markers of the osteogenic clusters found in our mouse analyses, including TRPV4, S100B, and Sox9, are present in ruptured human coronary lesions but not stable plaques (Figure 6C). Conversely, coronary lesions graded as stable were enriched for Phactr1 in the Acta2^+^ cells in the fibrous cap (Figure 6B). Taken together, our results suggest that most SMC phenotypes in advanced atherosclerotic lesions are conserved between humans and mice, and, taken with previous knowledge that SMC^Klf4^ is atheropromoting in mice and contains a human GWAS CAD risk locus, help us to infer that Klf4-dependent changes in SMC phenotype to an Lgals3^+^ state are likely detrimental for atherosclerosis pathogenesis.

## Discussion

Different SMC phenotypes have been hypothesized to have distinct roles in atherosclerotic lesion pathogenesis, some beneficial and some detrimental. In the present study, direct comparison of gene programs showed that SMC-specific knockout of Klf4 versus Oct4 resulted in nearly opposite genomic signatures consistent with the profoundly different plaque phenotypes exhibited by these mice (ie, smaller lesions with a thickened SMC-enriched fibrous cap for SMC^Klf4^ mice and larger lesions nearly lacking a SMC-enriched fibrous cap in SMC^Oct4^ mice).^3,4^ The results are of major interest, because they indicate that just 2 transcription factors that act cooperatively in regulating stem cell pluripotency^30^ paradoxically result in virtually opposite lesion phenotypes by altering the transcriptome of SMCs.

Our scRNA-seq experiments, the first scRNA-seq focused exclusively on atherosclerotic lesions, identified 14 clusters, of which 7 are derived from SMCs based on rigorous lineage tracing. Only 3 of these 7 would have been identified as SMCs using traditional marker gene expression. These results are consistent with our previous findings showing that >80% of SMC-derived cells in advanced lesions have lost expression of traditional markers.^3^ However, our results greatly extend those studies by defining the complexity of SMCs in late-stage lesions by the genes these cells express, including clusters defined by inflammatory markers (*Vcam1, Ccl2*), stem-like markers (*Ly6a*), production of ECM (*Lum, Dcn, Col1a1*), and osteogenic markers (*Runx2, Sox9, Cytl1*). Our analysis showing how SMC knockout of the CAD GWAS gene *Klf4* impacts these clusters provides several key insights about mechanisms whereby Klf4-dependent processes might exacerbate lesion pathogenesis, including not only SMC-macrophage transitions but also SMC-osteogenic transitions and a pioneer state marked by Lgals3. Altogether, expression of Klf4 is associated with negative effects on lesion pathogenesis that may be highly influential. Indeed, the *Klf4* network module in STARNET explains variance for CAD with a score >2, as large an effect size as low-density lipoprotein cholesterol.

Further, our studies allow us to reinterpret the meaning of Lgals3 in SMC phenotypic transitions in the context of atherosclerosis. In our original studies of mice with SMC-specific Klf4 knockout, we showed that approximately one third of Lgals3^+^ cells within mouse advanced BCA lesions were of SMC, not myeloid, origin, and that SMC-derived cells also express many additional markers of macrophages including CD11b and F4/80.^3^ Similar observations have been reported by many groups.^5,29,31–33^ The general assumption has been that a subset of SMCs within lesions have phenotypically modulated into a macrophage-like state that is negatively affecting lesion pathogenesis. Similar to Dobnikar et al,^33^ we did see some SMC-derived cells that expressed traditional macrophage markers CD11b and F4/80, but surprisingly, these were not the major population to change with Klf4 knockout in SMCs, although these cells may be undersampled by scRNA-seq.^20^ However, *Lgals3* was expressed in osteogenic clusters lost in SMC^Klf4^ mice, consistent with our original finding that Lgals3^+^ SMCs decrease in these mice. In addition to its role in macrophage differentiation and activation,^34,35^ Lgals3 has been shown to play a role in osteoblast differentiation^36^ and osteoclast function/recruitment,^37^ as well as SMC transition to an osteogenic phenotype in vitro.^38^ Interestingly, Lgals3 is necessary for transformation and maintaining stemness in some cancer cell lines^39–41^ and the migration of cancer cells and epithelial cells.^42,43^ Our scRNA-seq analysis also detected Lgals3^+^ activation in clusters with rich expression of matrix components consistent with observations by Wirka et al^7^ describing an Lgals3^+^ fibrocyte that they postulate plays a role in plaque stability. However, the ECM-matrix population decreases with SMC^Klf4^ knockout, and instead we see a 10-fold increase in SMC-derived Acta2^+^ Phactr2^+^ cells in the fibrous cap. In the natural course of bone formation, cartilaginous deposition of ECM components is a necessary predecessor to calcification,^44^ and it follows that matrix formation important for plaque stability might be continuous with the calcification process. Therefore, distinguishing SMC subsets that affect this process and their regulation is crucial for development of future therapies.

Our analysis also identified novel markers of SMC phenotypic clusters that can be identified at the transcript and protein level in both mouse models and human tissues. In particular, the congruence of our mouse and human scRNA-seq data offers hope that specific transcriptional signatures can be used to identify SMC-derived cells in humans without lineage tracing. We also found an intriguing overlap between our putative target genes in SMC^Klf4^ or SMC^Oct4^ murine atherosclerotic lesions and genes linked with nearly 40% of human CAD GWAS loci.^18^ For the majority of CAD GWAS loci, the mechanisms of action influencing the disease phenotype are completely or mostly unknown^18,19,45,46^ and reveal an auspicious depth of untapped potential for us to understand how CAD pathogenesis is influenced by SMC phenotype. In this work, we have shown that at least one third of the putative CAD target genes from murine lesions are altered by loss of Klf4 or Oct4 in cultured SMCs. However, we fully recognize that extensive further studies of each of these individual putative Klf4 and Oct4 target genes will be needed to show that they have a causal role in regulating SMC phenotype and CAD pathogenesis, including determining if CRISPR (clustered regularly interspaced short palindromic repeats)-Cas9–induced human GWAS risk alleles are associated with functional changes in SMCs that exacerbate lesion pathogenesis.^47^

Taken together, our results show that Klf4 regulates transition of SMCs to an Lgals3^+^ osteogenic phenotype, likely detrimental given our previous atheroprotective phenotype in SMC^Klf4^ mice. In addition, we provide novel evidence that diverse SMC lesion phenotypes are derived from a subset of Lgals3^+^ transitional state SMCs and show marked similarity to human plaque cells, suggesting this may be a rich area for development of novel therapeutics that promote beneficial versus detrimental changes in SMC phenotype.

## Acknowledgments

The authors thank the University of Virginia genomics core facility, including Dr Katia Sol-Church, Alyson Prorock, and Yongde Bao for assistance with single cell sequencing; the University of Virginia flow cytometry core facility, especially Michel Solga and Claude Chew for cell sorting assistance; and the University of Virginia advanced microscopy core, especially Stacey Guillot for imaging and Karen Soohoo for assistance with immunofluorescent imaging quantification.

## Sources of Funding

This work was supported by National Institutes of Health grants R01 HL132904, HL136314, and HL141425 to Dr Owens.

## Disclosures

None.

## Supplemental Materials

Data Supplement Methods

Data Supplement Tables I and II

Data Supplement Figures I–XVIII

References 48–52

Data Supplement Excel Files I and II

Data Supplement Movies I and II

## Supplementary Material


