# Supplementary material for: Stem Cell Pluripotency Genes Klf4 and Oct4 Regulate Complex SMC Phenotypic Changes Critical in Late-Stage Atherosclerotic Lesion Pathogenesis
Source: Circulation. 2020 Jul 17;142(21):2045–59. doi: 10.1161/CIRCULATIONAHA.120.046672 (PMC7682794; doi:10.1161/CIRCULATIONAHA.120.046672)
Supplement: Supplementary file 1 [file cir-142-2045-s001.pdf]

SUPPLEMENTAL MATERIALS

Supplemental Methods

PDGF scratch wound assay

SMC were isolated from aorta from our SMC lineage tracing mice (n=15) by enzymatic dissociation, sorted based on being eYFP+, and grown in DMEM F12 supplemented with penicillin/streptomycin, L-glutamine, and 10% fetal bovine serum (DF10). Flow cytometric analysis confirmed cultures were > 98% lineage traced SMC even at later passages. SMC cultures were treated with siLgals3 (SMARTpool Dharmacon, M-041097-01-005) or siNeg scramble control, serum starved for 24 hours, and scratched with a pipet tip and treated with 10 ug/ml PDGF-BB (Millipore, 01-305) in DF10 media. Light microscopy images were taken on an Olympus IX71 microscope at 10x zoom using cellSens standard software at three different points along the scratch, each at time 0, 24, and 48 hours. Results were quantified as the number cells that had entered the scratch area at time 0 hours, n=6.

Explant assay

Myh11-DreERT2 Rosa-tdTomato-eGFP and Myh11-DreERT2 Lgals3-Cre Rosa-tdTomato-eGFP mice were perfused with sterile saline and penicillin/streptomycin and aorta was removed using sterile tools. Aortae were digested in Liberase (Roche #355374) for 10 minutes at 37 degrees Celsius and the adventitia removed with sterile tools. Explants were plated on 6 well plates coated with 0.01% gelatin and incubated for 1 week in DF10 medium with 10% fetal bovine serum.

CyTOF sample preparation

Cell suspensions were prepared as described for scRNA-seq. Samples were washed with PBS, treated with 5μM cisplatin (Sigma Aldrich) for 30 seconds, quenched with Cell Staining Medium (CSM)(0.5% w/v Fraction V BSA (VWR) in PBS with 0.02% w/v sodium azide (Sigma Aldrich), washed again with PBS (Gibco), and fixed in 1.6% paraformaldehyde (Electron Microscopy Sciences) for 10 minutes with intermittent vortexing. Cells were washed into CSM and stored at -80C.

Samples were barcoded with palladium reagents and pooled[48]. The samples were washed with CSM and a single surface antibody cocktail (supplemental table) was prepared and added to the barcoded samples,

incubating at room temperature with shaking for 30 minutes. Barcoded samples were again washed with CSM and permeabilized with -20C 100% methanol (VWR), incubating for 10 minutes on ice and vortexing intermittently. After washing with CSM, samples were incubated in an intracellular antibody cocktail at room temperature with shaking for one hour. Samples were washed in CSM and incubated overnight at 4C with 1:5000 Iridium intercalator (Fluidigm) in 1.6% paraformaldehyde in CSM. Samples were washed with CSM and washed into water with normalization beads[49] and run on a Helios 2 mass cytometer (Fluidigm).

CyTOF data analysis

After measurement the samples were normalized[49], de-barcoded[50], and uploaded to Cytobank (www.cytobank.org) for cleanup gating before exporting FCS files for downstream analysis. Raw data was asinh transformed using a division factor of 5[51]. Dimensionality reduction was done by UMAP[52] using default parameters before using the full graph outputted by UMAP for Leiden clustering with default parameters.

Cell processing for scRNA-seq, mouse

Samples were minced and digested with 1mL Liberase (Roche, #355374) plus 1ug/mL ActinomycinD for 1 hour at 37 degrees, then resuspended in PBS plus 0.04% UltraPure non-Acetylated BSA (ThermoFisher #AM2616) and filtered through 30um filters on ice. Samples were then either counted and submitted directly for scRNAseq or sorted by flow cytometry. Samples for flow were stained with Sytox Blue viability dye and sorted with a BD FACSAria Fusion cell sorter with gating on live, single cells that were tdTomato+, GFP+, or eYFP+. Cells were collected into LoBind tubes containing 0.04% non-acetylated BSA.

Cell processing for scRNA-seq, human

Human carotid plaques were collected during CEA, with the time between surgical removal and plaque processing was less than 10 minutes. The remainder of the plaque washed in RPMI and minced and then digested in RPMI 1640 containing 2.5 mg/mL Collagenase IV (ThermoFisher Scientific), 0.25 mg/mL DNAse I (Sigma), 2.5 mg/mL Human Albumin Fraction V (MP

Biomedicals) and 1 mM Flavopiridol (Selleckchem) at 37oC for 30 minutes. Subsequently, the plaque cell suspension was filtered through a 70 µm cell strainer and washed with RPMI 1640.Cell viability was assed using Calcein AM and Hoechst (ThermoFisher Scientific) and sorted using the Beckamn Coulter MoFlo Astrios EQ.

Supplemental Movie I - Example I of explant of aorta showing loss of tdTomato in tdTomato+eGFP+ over time

Supplemental Movie II - Example II of explant of aorta showing loss of tdTomato in tdTomato+eGFP+ over time

Supplemental Excel File I - Pathway analysis of scRNA-seq clusters

Supplemental Excel File II - scRNA-seq differential gene expression of $SMC^{\mathit{Klf4}\text{-}\Delta/\Delta}$ vs $SMC^{\mathit{Klf4}\text{-WT/WT}}$

Methods Table I- Immunofluorescent staining antibodies

Primary Antibody	Catalog #	Concentration	Secondary antibody	Catalog #	Concentration
GFP	Abcam ab6673	1:250	donkey anti-goat AF488	Invitrogen A11055	1:250
			donkey anti-goat AF647	Invitrogen A21447	1:250
tdTomato	Rockland 600-401-379	1:100	donkey anti-rabbit AF546	Invitrogen A10040	1:250
Lgals3	Cedarlane CL8942AP	1:500	donkey anti-rat DyLight650	Abcam ab102263	1:250
TRPV4	Novus NB110-74960	1:250	donkey anti-rabbit AF647	Invitrogen A31573	1:250
S100b	GeneTex GTX129573	1:500	donkey anti-rabbit AF546	Invitrogen A10040	1:250
Sox9	Novus NBP1-85551	1:250	donkey anti-rabbit AF488	Invitrogen A21206	1:250
Phactr1	GeneTex GTX122251	1:100	donkey anti-rabbit AF488	Invitrogen A21206	1:250
			donkey anti-rabbit AF647	Invitrogen A31573	1:250
Acta2-Cy3	Sigma Aldrich C6198	1:500 of 1.2 mg/mL	n/a		1:250
Acta2-FITC	Sigma Aldrich F3777	1:500 of 2.2mg/mL	n/a		1:250
Vcam1	GeneTex GTX53135	1:100	donkey anti-rat AF488	Invitrogen A21208	1:250
Spp1/Osteopontin	Novus NB600-1043	1:500	donkey anti-rabbit AF647	Invitrogen A31573	1:250
Ly6a/Sca1	Abcam ab51317	1:200	donkey anti-rat DyLight650	Abcam ab102263	1:250

Methods Table II- CyTOF antibodies

Metal	Marker	Clone	Vendor	Staining Concentration	Surface or Intracellular
Y89	CD45	30-F11	Fluidigm	120ng/mL	Surface
In115	CD106	429 (MVCAM.A)	BD	1600ng/mL	Surface
La139	CD206	C068C2	Biolegend	600ng/mL	Intracellular
Pr141	CD140b	APB5	eBioscience	300ng/mL	Surface
Nd142	TRPV4	Polyclonal	Invitrogen	600ng/mL	Intracellular
Nd143	CD117	2B8	Biolegend	1000ng/mL	Surface
Nd145	Desmin	RD301	Abcam	2000ng/mL	Intracellular
Nd146	S100B	4C4.9	Abcam	10000ng/mL	Intracellular
Sm147	CD200	OX-90	Biolegend	300ng/mL	Surface
Nd148	NG2	546930	R&D	2000ng/mL	Surface
Nd150	Oct3/4	40/Oct-3	BD	2000ng/mL	Intracellular
Eu151	RFP	Polyclonal	Abcam	2000ng/mL	Intracellular
Sm154	Runx2	232902	R&D	1000ng/mL	Intracellular
Gd155	CD31	390	Biolegend	7.5ng/mL	Surface
Gd156	CD184	L276F12	Biolegend	300ng/mL	Surface
Gd157	CD124	mIL4R-M1	BD	30ng/mL	Surface
Gd158	CD93	AA4.1	Fluidigm	0.1nL/200uL	Surface
Tb159	SMA	1A4	eBioscience	4ng/mL	Intracellular
Dy161	CD34	Ram34	eBioscience	250ng/mL	Surface
Dy162	GFP	338002	Biolegend	4000ng/mL	Intracellular
Dy163	CD11b	M1/70	Biolegend	250ng/mL	Surface
Dy164	CX3CR1	SA011F11	Fluidigm	0.1uL/200uL	Surface
Er167	IL-6	MP5-20F3	Fluidigm	1uL/200uL	Surface
Er168	CD140a	APA5	Biolegend	1000ng/mL	Surface
Tm169	Ly6A/E	D7	Fluidigm	0.1uL/200uL	Surface
Er170	IFNg	XMG1.2	Biolegend	10000ng/mL	Intracellular
Yb171	CD44	IM7	BD	1000ng/mL	Surface
Yb172	CD86	GL1	Fluidigm	1uL/200uL	Surface
Yb173	Cl. Casp3	C92-605	BD	500ng/mL	Intracellular
Yb174	Klf4	Polyclonal	CST	2000ng/mL	Intracellular
Lu175	F4/80	BM8	Biolegend	360ng/mL	Surface

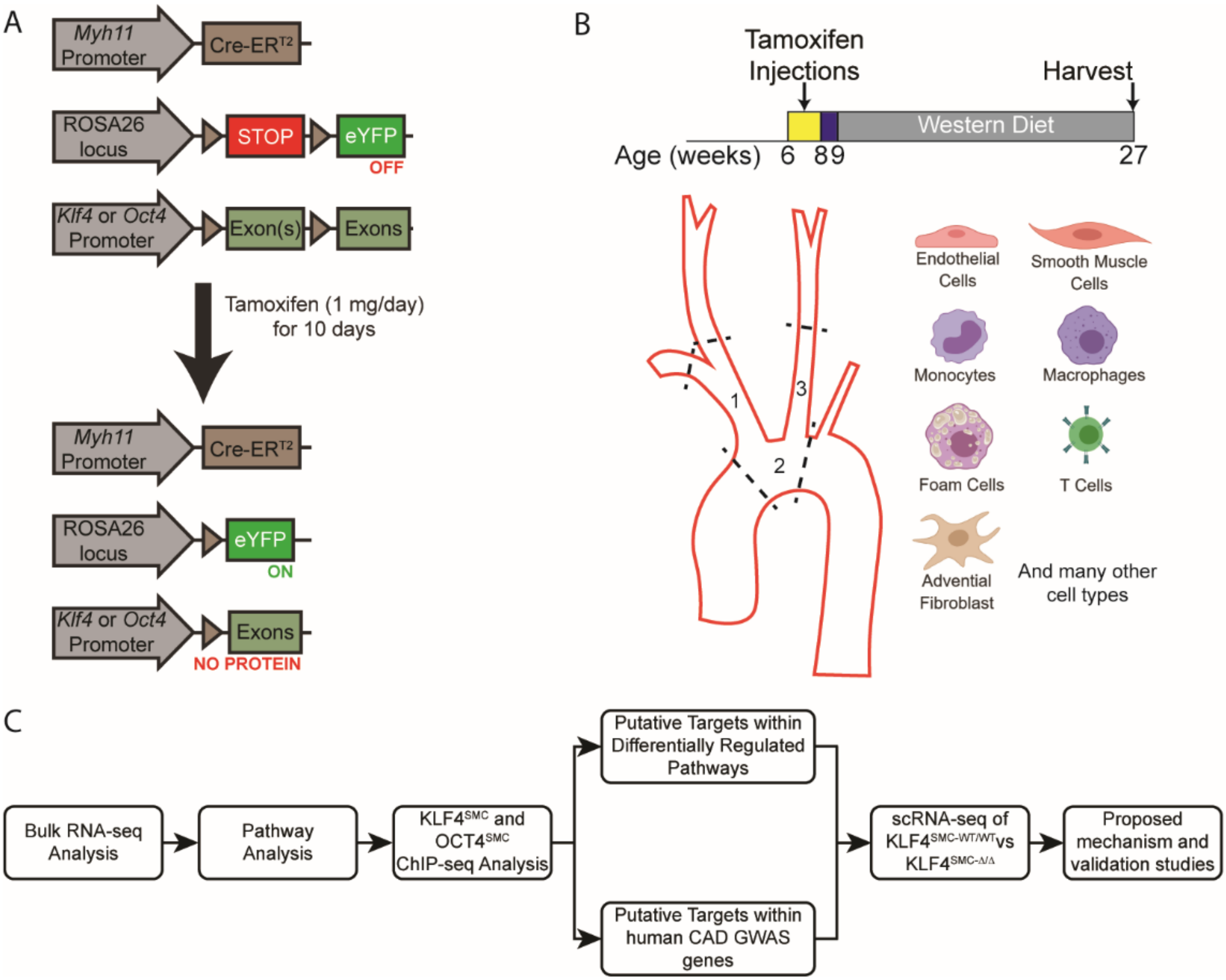

Supplemental Figure I. **Mouse model and genomic strategies to identify putative factors/genes that regulate plaque stability.** A) Design of SMC lineage tracing mice, where the SMC-specific Myh11 promoter-enhancer region is used to drive a tamoxifen inducible Cre. This Cre will excise a STOP codon flanked by loxP sites in the Rosa26 locus and label all cells that express *Myh11*(SMCs and pericytes) at the time of tamoxifen injection with eYFP as well as the progeny of these cells. It will also further remove exons located in either the *Klf4* or *Oct4* loci, knocking out that specific gene in SMCs. B) Mice were injected with tamoxifen at 6-8 weeks of age to activate the Myh11-Cre^{ERT2} and label all SMC expressing *Myh11* at that time with eYFP. After a week of recovery, mice are put on western diet for 18 weeks to induce late stage atherosclerotic lesions in the brachiocephalic artery (BCA). The BCA region, with part of the left carotid and aortic arch, was harvested for RNA-seq and ChIP-seq. C) Analysis strategy to find potential targets of *Klf4* and *Oct4* in SMC that contribute to the atherosclerosis phenotype observed with SMC$^{Klf4\text{-}\Delta/\Delta}$ and SMC$^{Oct4\text{-}\Delta/\Delta}$ ApoE$^{-/-}$ knockout mice. scRNAseq analyses were done on the advanced BCA lesions alone with pooling of micro-dissected lesion samples from 4-6 mice for each experimental group for each independent experiment.

A

Genotype	Up-regulated GSEA KEGG Pathways (FDR q-val < 0.05)	Down-regulated GSEA KEGG Pathways (FDR q-val < 0.05)
Klf4 KO vs WT	6	41
Oct4 KO vs WT	65	9

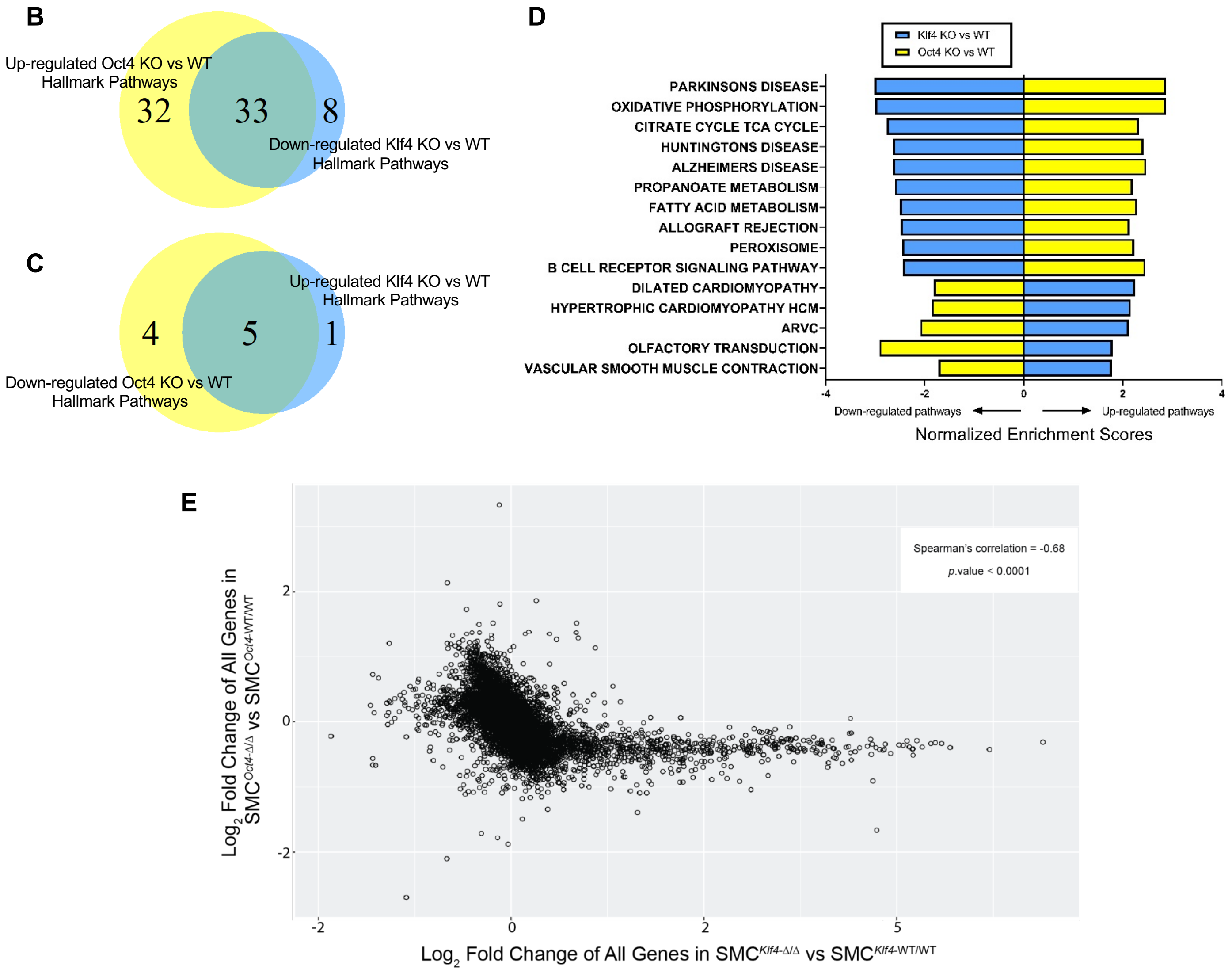

Supplemental Figure II. **Genomic analyses of the BCA region of SMC$^{Klf4\text{-}\Delta/\Delta}$ and SMC$^{Oct4\text{-}\Delta/\Delta}$ mice versus their respective littermate controls exhibited nearly opposite patterns of gene expression.** A) RNAseq on SMC$^{Klf4\text{-}\Delta/\Delta}$ vs SMC$^{Klf4\text{-}WT/WT}$ and SMC$^{Oct4\text{-}\Delta/\Delta}$ vs SMC$^{Oct4\text{-}WT/WT}$ advanced BCA lesions were analyzed for MSigDB KEGG pathways (FDR q value of 0.05). B-C) Pathways that were inversely regulated by SMC^{Klf4} versus SMC^{Oct4} are shown in B (down-regulated in Klf4 model and up-regulated in Oct4 model) and C (inverse of B) D) All common KEGG pathways in C and the top 8 common dysregulated pathways are shown. Normalized enrichment scores show downregulated pathways on the left and up regulated pathways on the right. E) Analysis of global gene expression profiles between advanced BCA lesions of SMC$^{Klf4\text{-}\Delta/\Delta}$ vs SMC$^{Klf4\text{-}WT/WT}$ and SMC$^{Oct4\text{-}\Delta/\Delta}$ vs SMC$^{Oct4\text{-}WT/WT}$ mice showed a significant negative correlation. Every dot corresponds to a gene. The X-axis is the $\log_2$ fold change of SMC$^{Klf4\text{-}\Delta/\Delta}$ vs SMC$^{Klf4\text{-}WT/WT}$, and the Y-axis is the $\log_2$ fold change of SMC$^{Oct4\text{-}\Delta/\Delta}$ vs SMC$^{Oct4\text{-}WT/WT}$ for each gene identified in our bulk RNAseq analyses of the BCA regions.

A

Ingenuity Canonical Pathways	-log(p-value)
Remodeling of Epithelial Adherens Junctions	17.6
Epithelial Adherens Junction Signaling	16.5
Integrin Signaling	14.0
Germ Cell-Sertoli Cell Junction Signaling	13.9
Axonal Guidance Signaling	13.6
RhoGDI Signaling	13.6
Regulation of Actin-based Motility by Rho	13.2
Actin Cytoskeleton Signaling	10.8
Leukocyte Extravasation Signaling	9.9
ILK Signaling	9.49

B

Ingenuity Canonical Pathways	-log(p-value)
Role of NANOG in Mammalian Embryonic Stem Cell Pluripotency	6.87
Axonal Guidance Signaling	6.73
Human Embryonic Stem Cell Pluripotency	6.27
Glioblastoma Multiforme Signaling	5.94
Molecular Mechanisms of Cancer	5.75
Role of Macrophages, Fibroblasts and Endothelial Cells in Rheumatoid Arthritis	5.73
Regulation of the Epithelial-Mesenchymal Transition Pathway	5.63
Signaling by Rho Family GTPases	5.55
Neuropathic Pain Signaling In Dorsal Horn Neurons	5.31
CREB Signaling in Neurons	5.21

E

SMC$^{Oct4\text{-}\Delta/\Delta}$ vs SMC$^{Oct4\text{-}WT/WT}$

Gene Name	log2FC	padj
Arhgef26	-4.55	7.90E-97
Atp5g1	-0.37	0.006
Coro6	-1.84	0.001
Dst	-0.415	3.40E-04
Edn1	0.5899	0.01
Irs1	1.91	2.93E-50
Mad2l1	0.733	0.001
Map3k1	0.842	9.93E-22
Msl2	0.389	3.90E-04
Phactr1	-1.54	0.003
Plekhg1	-1.79	6.20E-05
Procr	-1.56	1.12E-39
Sf3a3	0.67	1.05E-10
Shisa4	-0.425	0.006
Slc22a4	1.15	0.004
Ssh2	-0.413	0.002
Swap70	-0.297	0.025
Tom1l2	-0.431	3.58E-04
Top1	0.599	3.61E-10
Trib1	-0.997	4.15E-14
Zeb2	0.775	1.15E-15

C

MSigDB Pathway	Binomial FDR
Focal adhesion	8.37e-83
Beta1 integrin cell surface interactions	1.33e-74
Genes involved in Extracellular matrix organization	5.90e-72
ECM-receptor interaction	2.47e-70
Genes involved in Collagen formation	1.27e-64
Genes involved in Integrin cell surface interactions	3.27e-62
Genes involved in NCAM1 interactions	4.72e-59
Genes involved in NCAM signaling for neurite out-growth	2.58e-56
Syndecan-1-mediated signaling events	6.64e-55
Genes involved in Platelet activation, signaling and aggregation	5.66e-54
Genes involved in Signaling by PDGF	1.81e-52
Genes involved in Platelet Adhesion to exposed collagen	2.68e-52
Genes involved in Axon guidance	1.84e-49
Integrins in angiogenesis	1.09e-45
Beta3 integrin cell surface interactions	5.21e-45

D

MSigDB Pathway	Binomial FDR
Vibrio cholerae infection	1.06e-55
Tryptophan metabolism	1.92e-18
Genes involved in RNA Polymerase III Transcription Initiation From Type 2 Promoter	1.39e-12
Genes involved in RNA Polymerase III Chain Elongation	1.15e-12
RNA polymerase	3.82e-9
Genes involved in RNA Polymerase III Transcription Initiation From Type 3 Promoter	1.48e-8
Taurine and hypotaurine metabolism	2.93e-8
Genes involved in RNA Polymerase III Transcription Termination	7.34e-8
Genes involved in Deposition of New CENPA-containing Nucleosomes at the Centromere	6.54e-8
Genes involved in RNA Polymerase III Transcription	6.93e-7
Genes involved in RNA Polymerase I, RNA Polymerase III, and Mitochondrial Transcription	3.62e-5
Regulation of autophagy	8.26e-5
Proteasome Complex	5.17e-4
Cytosolic DNA-sensing pathway	2.02e-3

F

SMC$^{Klf4\Delta/\Delta}$ vs SMC$^{Klf4\text{-}WT/WT}$

Gene Name	log2FC	padj
Apob	4.45	0.015
Arhgef26	-2.98	4.68E-05
Flt1	-2.4	0.004
Prim2	-0.997	0.007
Slc5a3	-3.6	0.019
Ump5	0.76	0.038

G

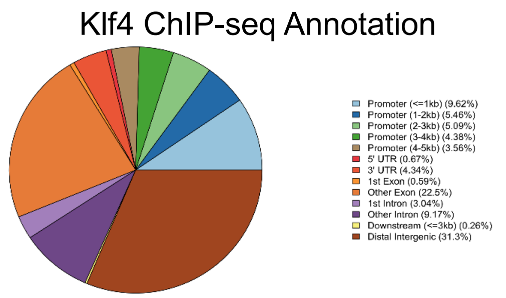

H

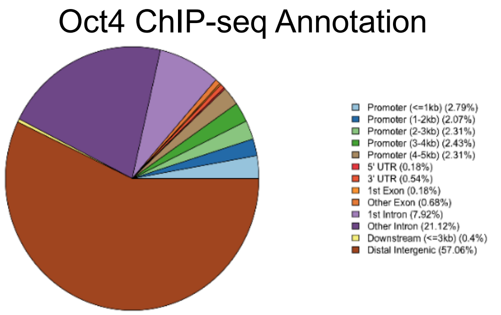

I

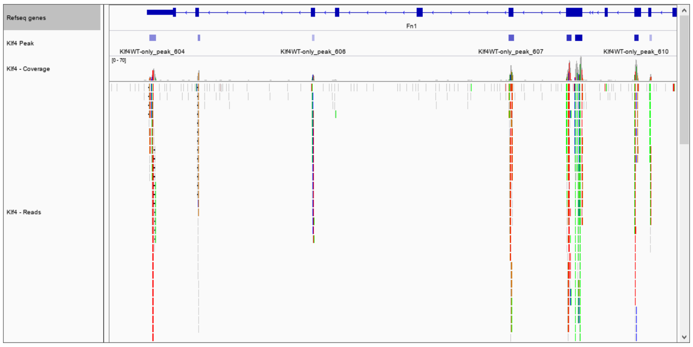

J

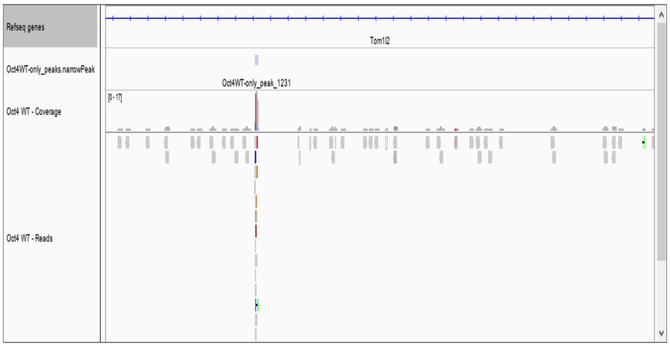

Supplemental Figure III. **Pathway analysis of genes identified in SMC$^{Klf4\text{-}WT/WT}$ vs $^{Klf4\text{-}\Delta/\Delta}$ and SMC$^{Oct4\text{-}WT/WT}$ vs $^{Oct4\text{-}\Delta/\Delta}$ ChIP-seq analysis and *in vitro* analysis of genes associated with CAD.** A-B) IPA canonical pathway analysis of putative *Klf4* (A, blue) or *Oct4* (B, yellow) target genes in SMC based on detection of DNA sequences enriched in ChIP-Seq analysis of BCA regions of SMC$^{Klf4\text{-}WT/WT}$ versus SMC$^{Klf4\text{-}\Delta/\Delta}$ and SMC$^{Oct4\text{-}WT/WT}$ versus SMC$^{Oct4\text{-}\Delta/\Delta}$ mice fed a western diet for 18 weeks. Genes were either present only on the WT animals and/or were significantly enriched in the WT compared to KO animals. C-D) Reactome pathway analysis of *Klf4* (C, blue) and *Oct4* (D) of putative binding targets in SMC shows similar pathways as A). E-F) *in vitro* (SMC$^{Klf4\text{-}WT/WT}$ versus SMC$^{Klf4\text{-}\Delta/\Delta}$ cholesterol loading experiment) and SMC$^{Oct4\text{-}WT/WT}$ versus SMC$^{Oct4\text{-}\Delta/\Delta}$ (hypoxia + POVPV) validation of CAD GWAS genes present in Figure 1 E-F. G-H) Annotation of Klf4 (G) and Oct4 (H) ChIP-seq peaks. I-J) Examples of Klf4 and Oct4 ChIP-seq peaks

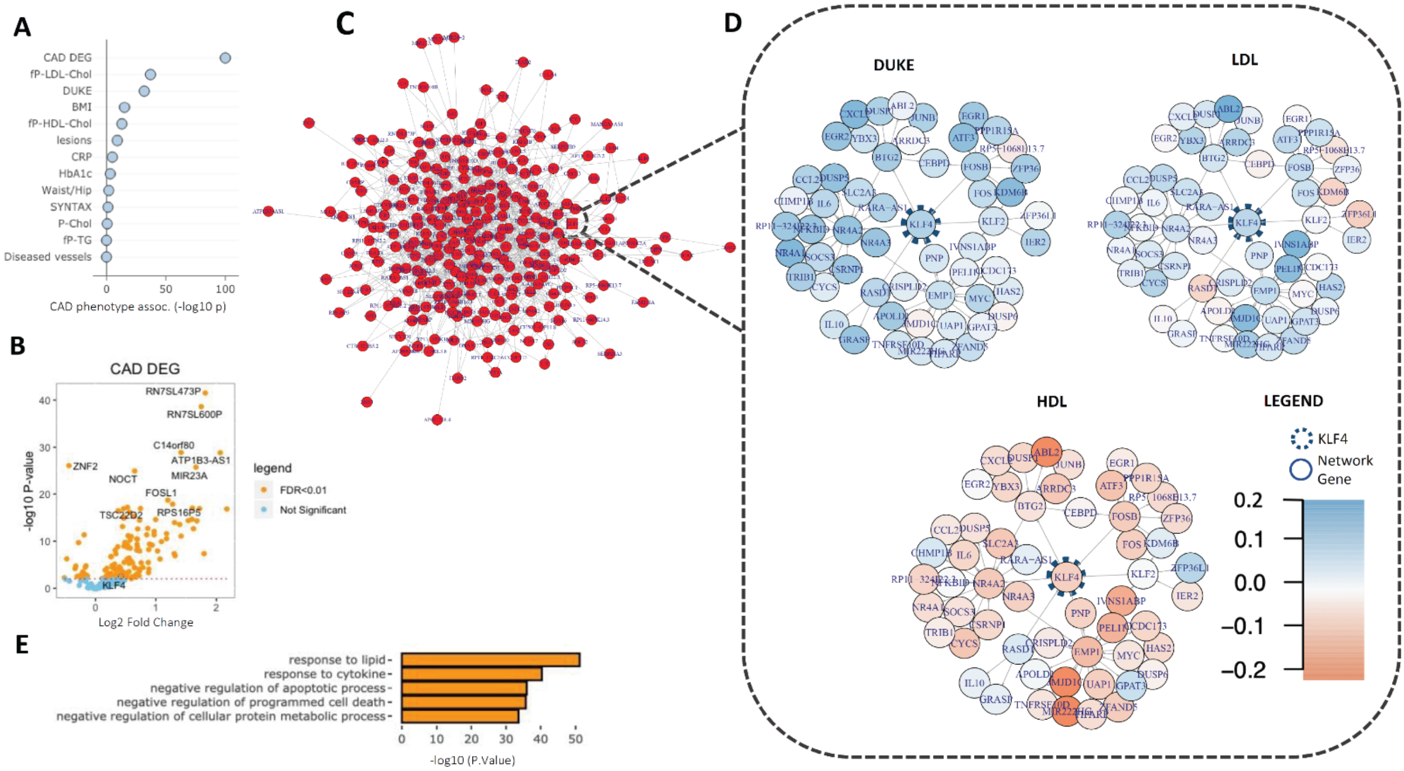

Supplemental Figure IV. **The atherosclerotic arterial wall STARNET co-expression module 33 harbors *KLF4.*** A) Bar chart showing the statistical significance (x-axis) of associations between the eigengene value of the STARNET co-expression module 33 and CAD-related phenotypes in STARNET (Y-axis). B) Volcano plot showing differentially expressed module 33 genes (i.e., CAD DEG in panel A) in the arterial wall of STARNET patients with obstructive CAD compared to non-CAD controls. C) Co-expression network module 33 in the atherosclerotic arterial wall (n=273). D) Network module 33 zoom-in of *KLF4* and its first and second network neighboring genes. Color coding indicate genes positively (red) and negatively (blue) associated with clinically significant extent of coronary atherosclerosis (i.e., *SYNTAX score*[3]), plasma levels of LDL (low density lipoprotein) and HDL (high density lipoprotein). E) Bar chart showing the statistical significance (x-axis) of top biological processes according to Gene Ontology enriched by module 33 genes indicating this module responds to plasma lipids and cytokines and is involved in regulating apoptosis.

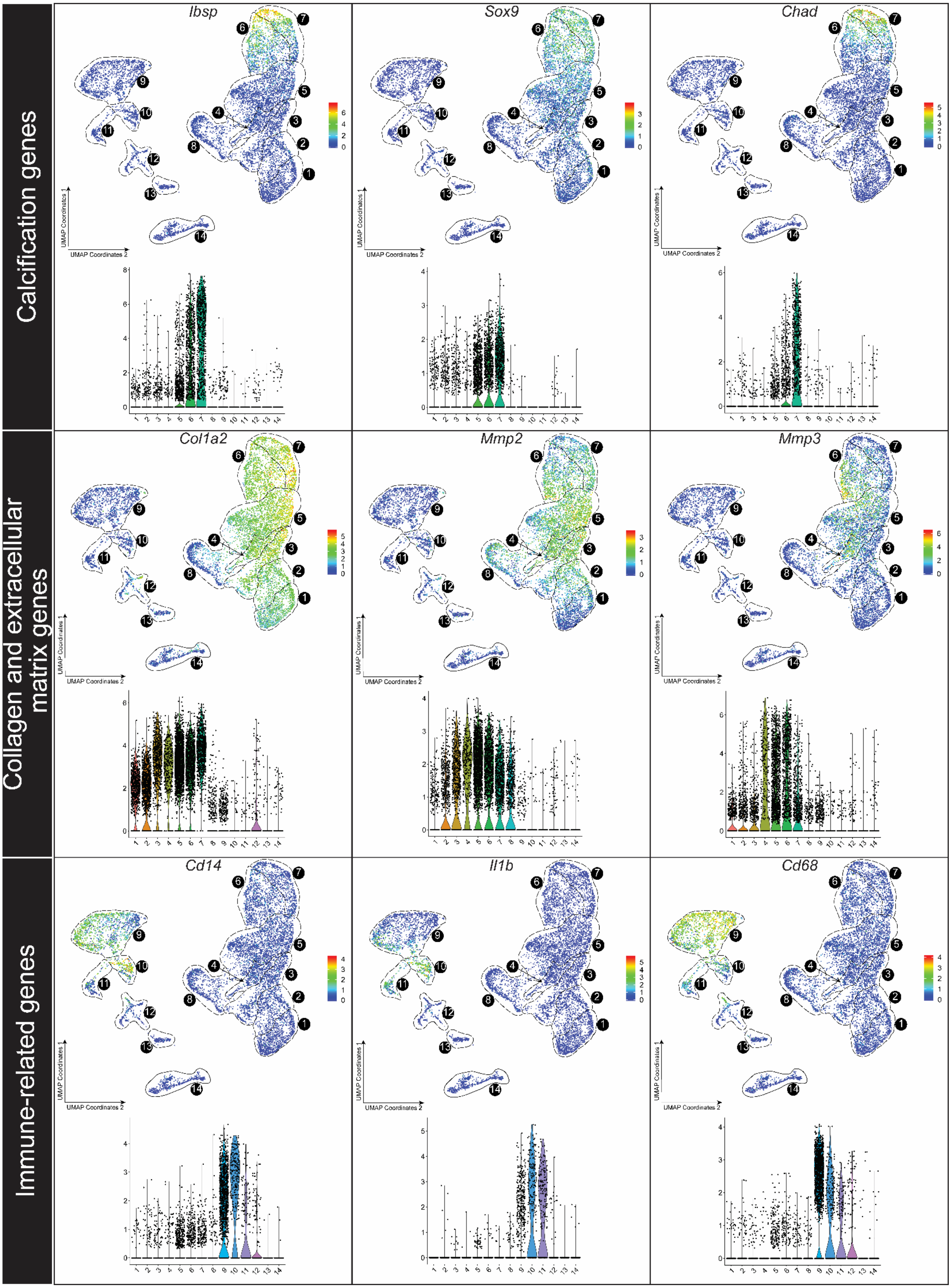

Supplemental Figure V. **UMAP showing genes involved in calcification (top row), collagen and extracellular matrix (middle row), and immune-related genes (bottom row) in scRNAseq of advanced mouse BCA lesions**. The top row shows the following calcification UMAP genes: *Ibsp*, *Sox9*, and *Chad*. These genes are enriched in cluster 13-15. The middle row shows the following collagen and extracellular matrix UMAP genes: *Col1a2*, *Mmp2*, and *Mmp3*. These genes are enriched in cluster 3-6. The bottom row shows the following immune-related UMAP genes: *Cd14*, *Il1b*, and *CD68*. These genes are enriched in cluster 9-11.

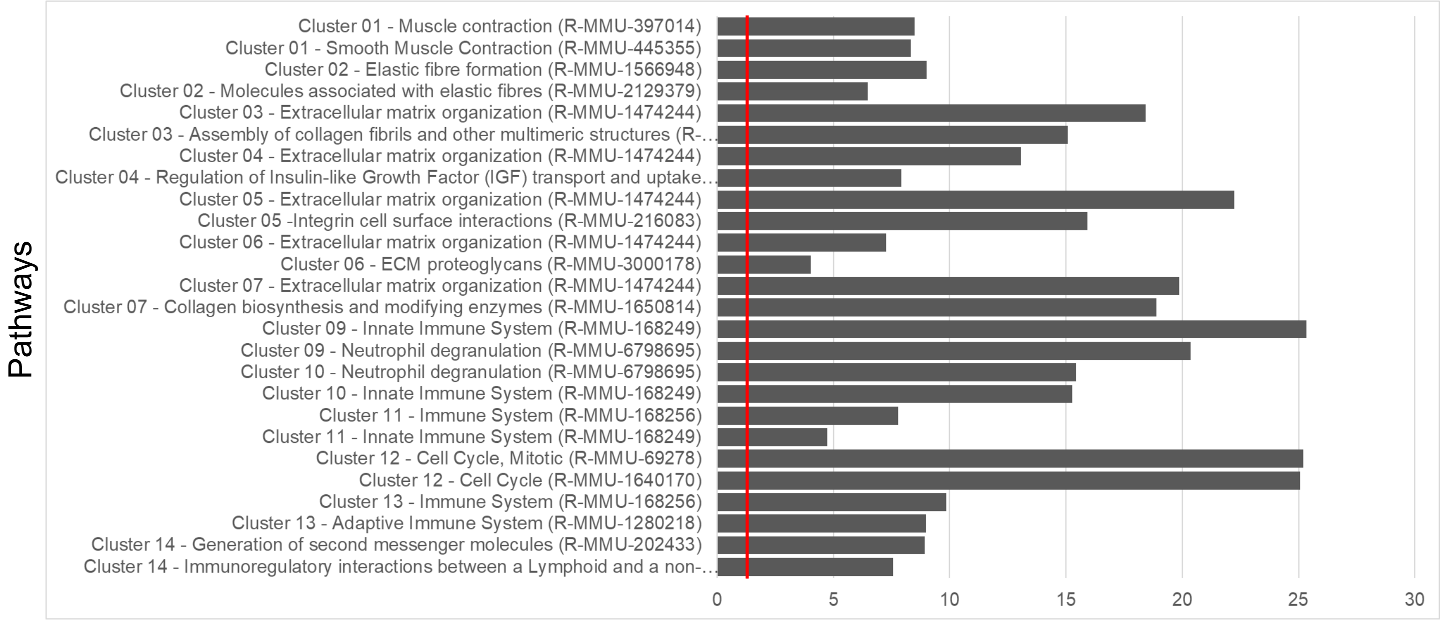

Supplemental Figure VI. **Pathway analysis of each cluster shows involvement in smooth muscle cell contraction, extracellular matrix production, and immune system.** Bar graph showing the most significant pathway associated with each cluster using the top 100 genes. Red stripped line indicates –log10 of 0.05 p-value. An Excel sheet is available with all the significant GO biological process and Reactome significant pathways for each cluster. Excel file I shows all the significant GO biological process and Reactome significant pathways for each cluster.

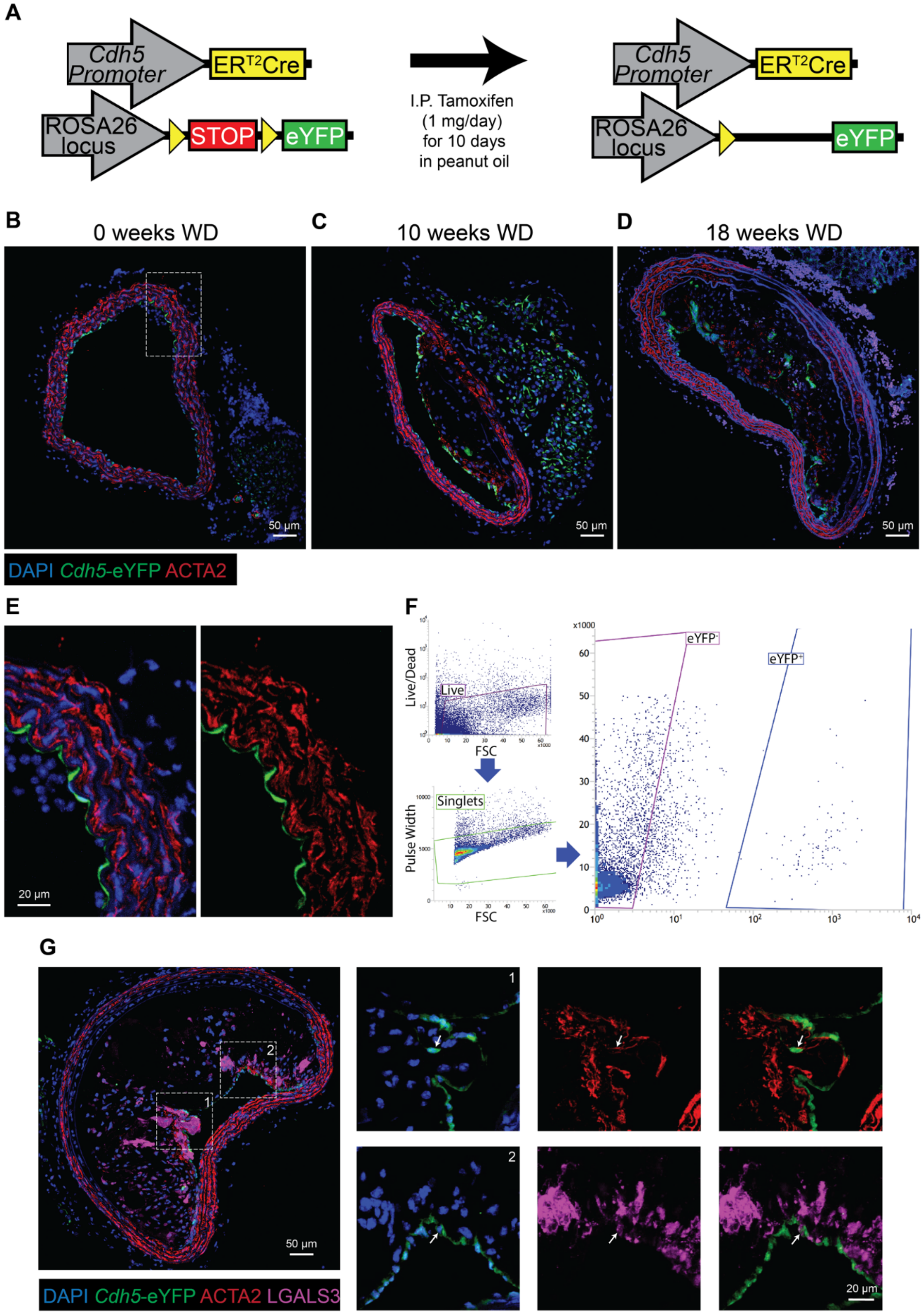

Supplemental Figure VII. **Cdh5-CreERT2 lineage tracing accurately labels endothelial cells in atherosclerosis.** A) Design of EC lineage tracing mice, where the Cdh5 promoter drives a tamoxifen inducible Cre recombinase. This Cre will excise a STOP codon flanked by FloxP sites in the Rosa26 locus and label all cells expressing Cdh5 (endothelial cells) and their progeny with eYFP at the time of tamoxifen injection. Mice were injected using the same experimental strategy described in Figure I of the Supplement. B-D) Cdh5-CreERT2 ApoE$^{-/-}$ mice were injected with tamoxifen from 6-8 weeks and then harvested at 0, 10, or 18 weeks of Western Diet. E) Enlarged area from panel B demonstrating endothelial cell-specific labeling with eYFP and no staining of the ACTA2+ medial cells. F) Gating strategy used to sort BCA lesions from Cdh5-CreERT2 ApoE$^{-/-}$ mice fed 18 weeks of WD for eYFP^{+} endothelial cells that were then submitted for 10X scRNA sequencing. G) Representative image of a Cdh5-CreERT2 ApoE$^{-/-}$ fed 18 weeks of WD with subset images highlighting examples of endothelial cells expressing either Acta2 or Lgals3.

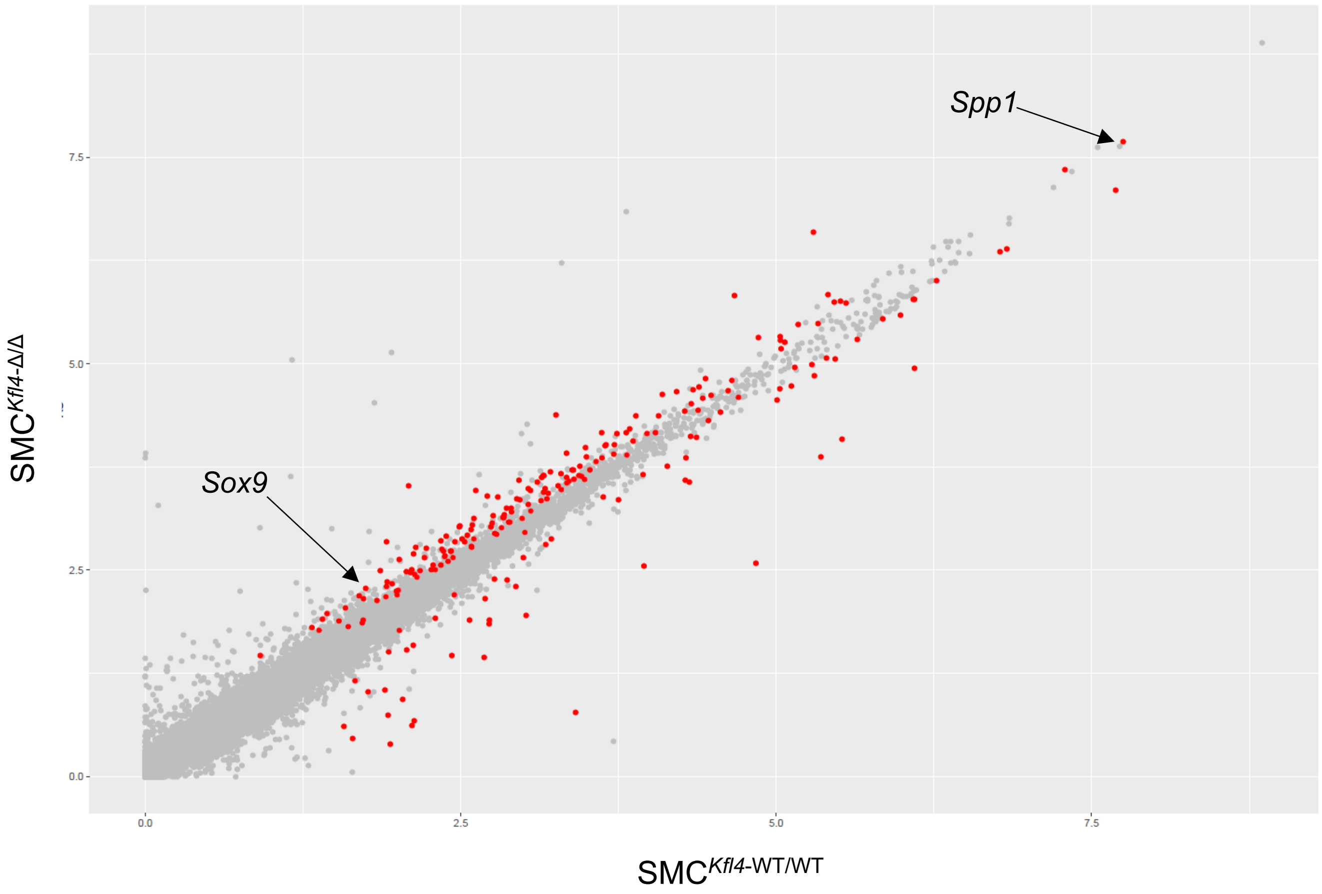

Supplemental Figure VIII. **Differential expression analysis of all SMC$^{Klf4\text{-}\Delta/\Delta}$ vs SMC$^{Klf4\text{-WT/WT}}$ cells show more than 80 differentially expressed genes, including down-regulation of *Sox9* and *Spp1* in SMC$^{Klf4\text{-}\Delta/\Delta}$.** Average expression for each gene across all lesion cells from SMC$^{Klf4\text{-}\Delta/\Delta}$ (Y-axis) or SMC$^{Klf4\text{-WT/WT}}$ (X-axis) scRNAseq of advanced BCA lesions is plotted to show differential expression of genes overall between SMC$^{Klf4\text{-}\Delta/\Delta}$ and SMC$^{Klf4\text{-WT/WT}}$.

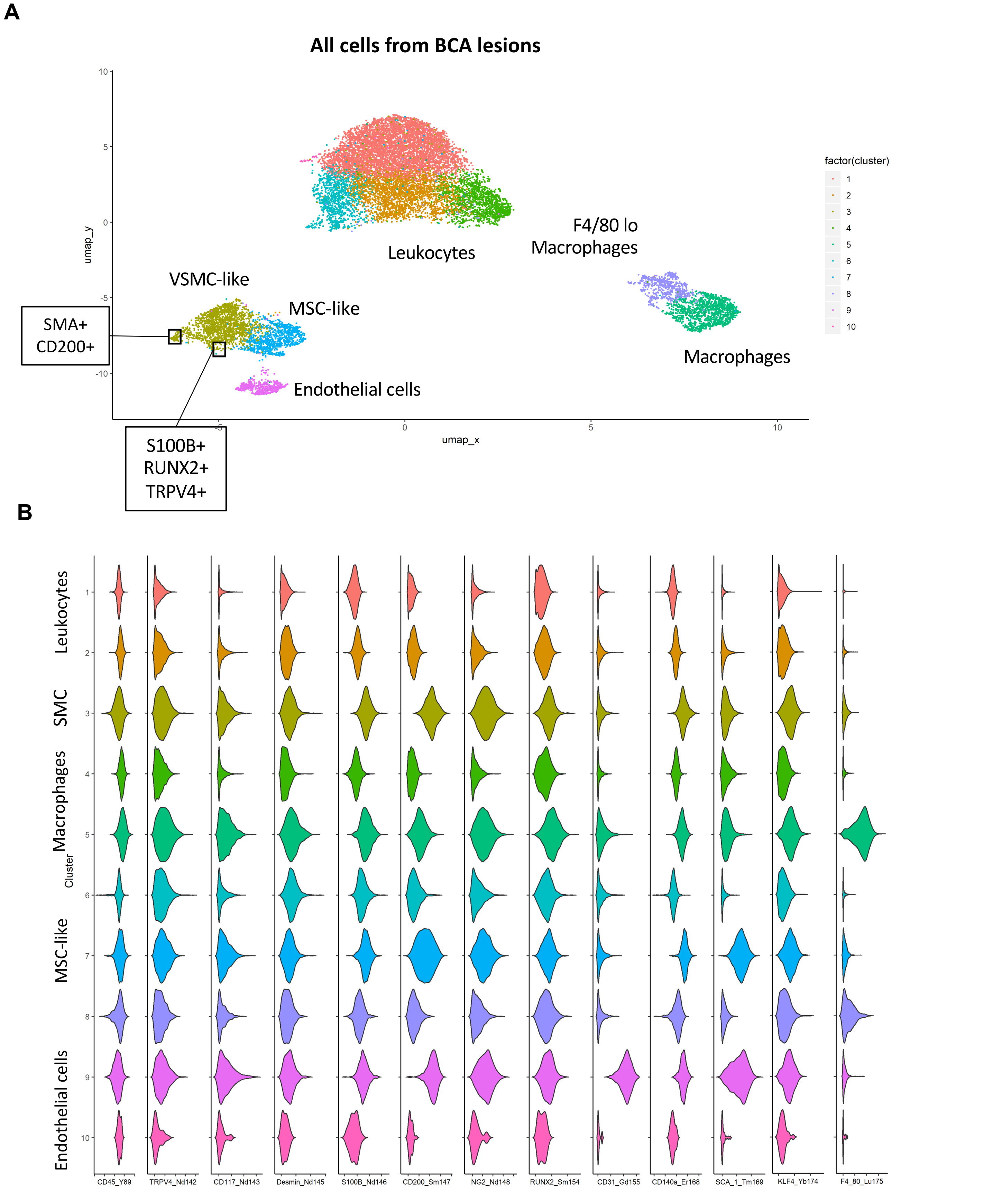

Supplemental Figure IX. **CyTOF of selected markers in advanced BCA lesion cells from SMC$^{Klf4\text{-}\Delta/\Delta}$ vs SMC$^{Klf4\text{-}WT/WT}$ animals.** A) Mass cytometric (CyTOF) results using a 31-marker panel was performed on advanced BCA lesions from SMCKlf4-WT/WT and SMCKlf4-Δ/Δ mice after 18 weeks of western diet. UMAP colored by cluster, highlighting areas with cells positive for markers identified by scRNAseq. B) Violin plots for detection of selected markers, each row represents a cluster and each column represents a marker in the CyTOF panel, showing marker expression of each cluster.

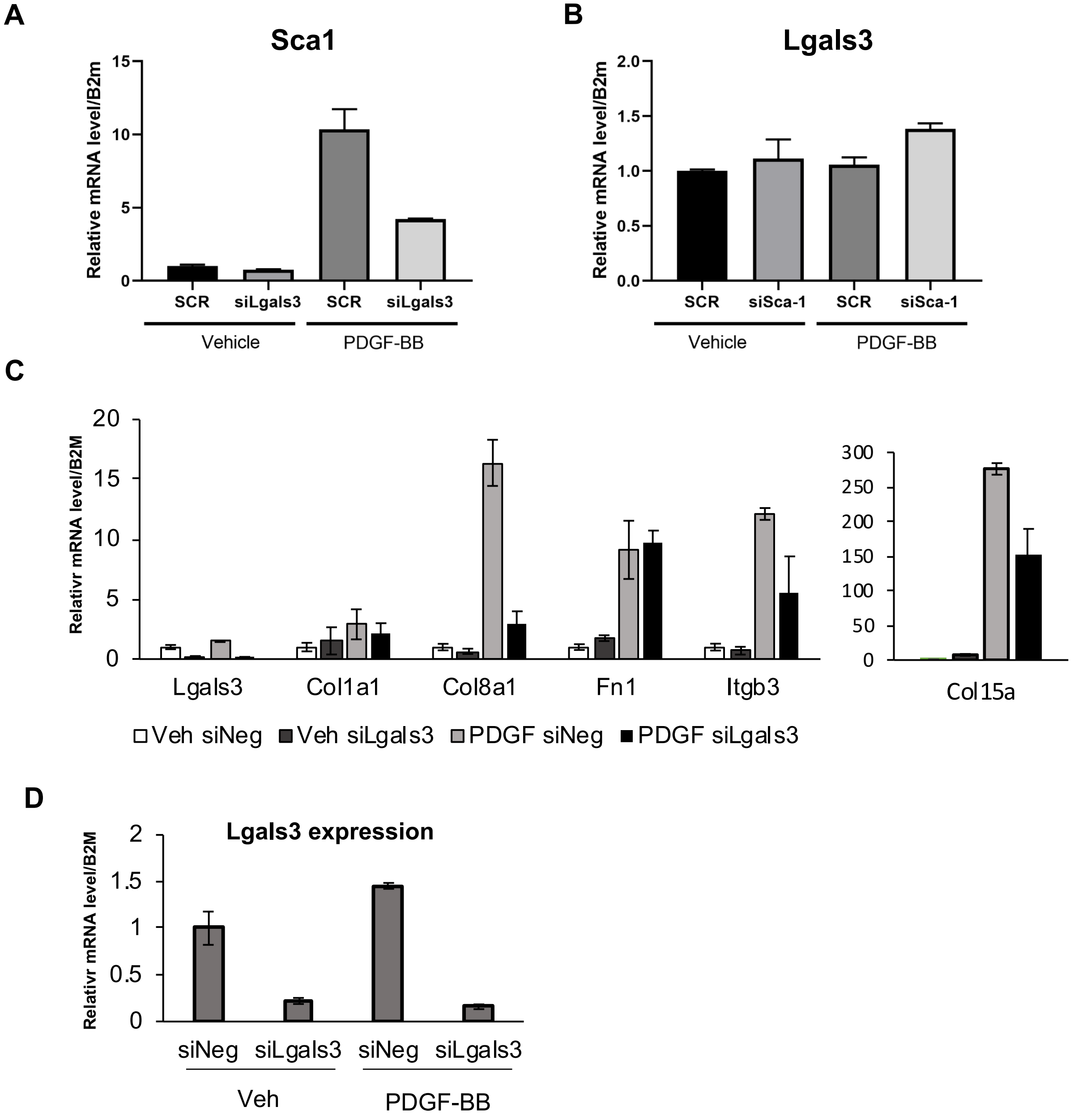

Supplemental Figure X. **siRNA-induced knockdown of Lgals3 in cultured mouse aortic SMC significantly decreased expression of Sca1 and multiple collagens.** A) SMC isolated from aorta from our SMC lineage tracing mice were treated with siLgals3, or siNeg scramble control, serum starved for 24 hours, and treated with 10 ug/ml PDGF-BB in DF10 media with 10% serum, n=3 biological replicates per condition. RNA was isolated and reverse transcribed, and expression of Sca1 was measured. Results are shown as ratio of expression over housekeeping gene B2M, with error shown as standard deviation between biological replicates. This experiment was repeated three times. B) Lineage traced SMC were treated with siSca1 or siNeg scramble control, serum starved for 24 hours, treated with PDGF-BB, and assayed for Lgals3 expression. C)Expression of various collagens was measured after siLgals3 knockdown cells treated with PDGF-BB from A). D) Knockdown efficiency from experiments in A).

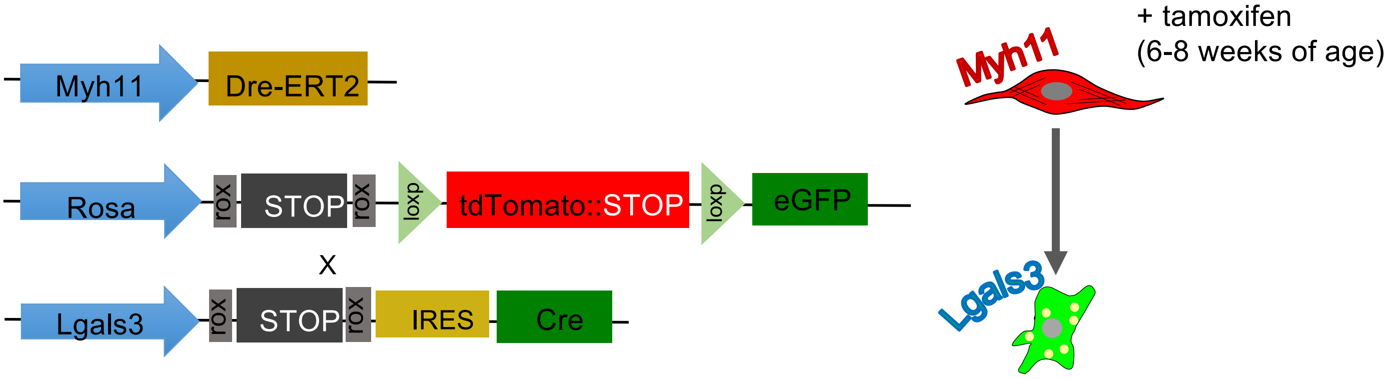

1. Myh11-expressing smooth muscle cells and pericytes are labeled with tdTomato upon activation of conditional, specific Dre recombinase by tamoxifen injection at 6-8 weeks of age
2. Dre unlocks an IRES Cre on the Lgals3 locus
3. Cre is activated only in SMC that sequentially express Lgals3, turning transitioned cells from red to green and generating a specific genetic knockout

Supplemental Figure XI. **Dual lineage tracing system design.** Myh11-expressing smooth muscle cells and pericytes are labeled with tdTomato upon activation of conditional, specific Dre^{ERT2} recombinase by tamoxifen injection at 6-8 weeks of age. Dre^{ERT2} unlocks an IRES Cre on the Lgals3 locus. Cre is activated only in SMC that sequentially express Lgals3, turning transitioned cells from red to green and generating a specific genetic knockout. Video describing this mouse can be found at https://www.cvrc.virginia.edu/Owens/educationaltopics.html.

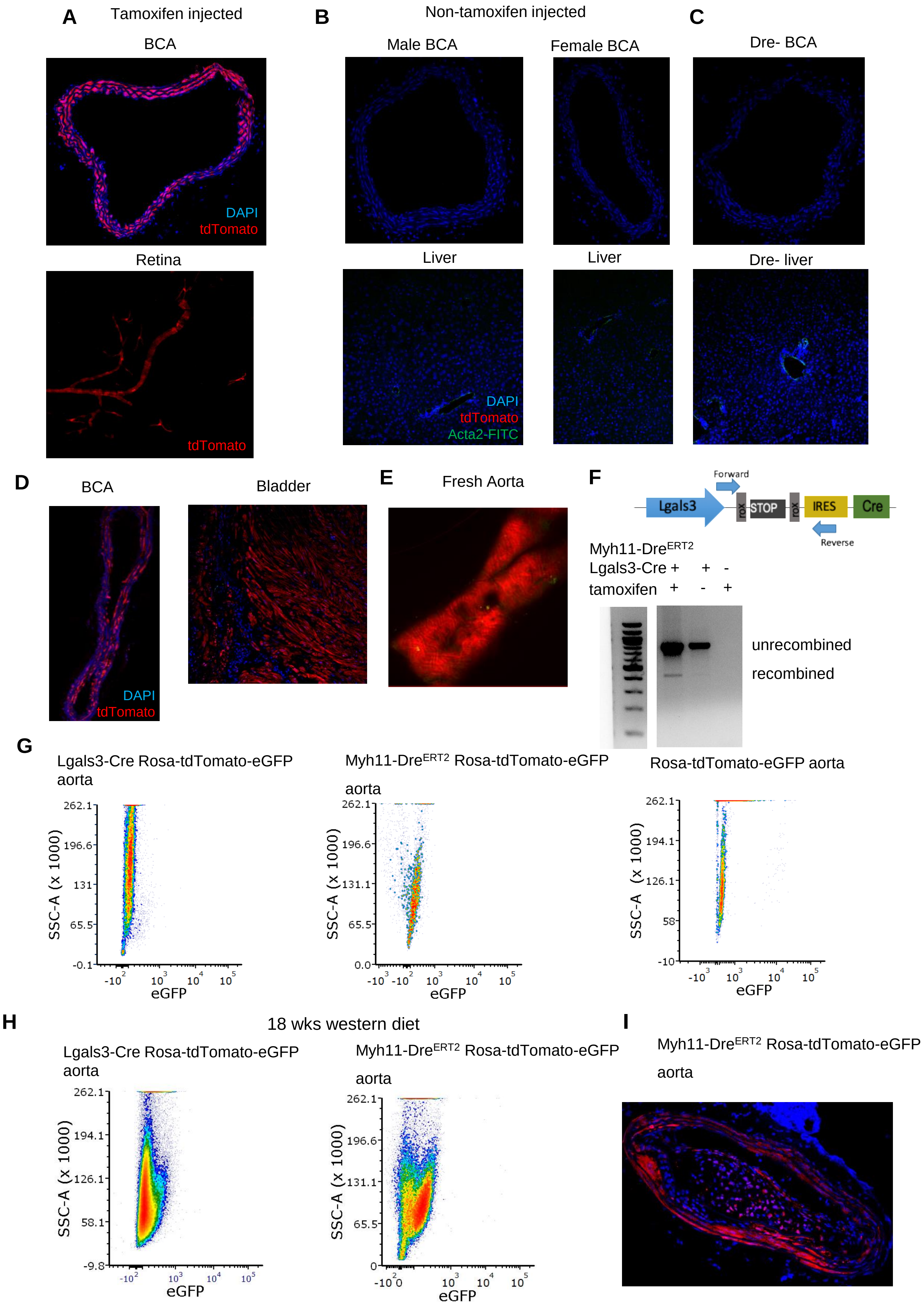

Supplemental Figure XII. **Dual lineage tracing system validation.**

Supplemental Figure XII. **Dual lineage tracing system validation**. A) Myh11-Dre^{ERT2} Rosa-tdTomato-eGFP mice were injected or not injected with tamoxifen at 6-8 weeks of age and various tissues subsequently harvested. Mice treated with tamoxifen (A) show efficient labeling of the brachiocephalic artery (BCA) and microvasculature (retina). Images were taken by confocal fluorescence microscopy of endogenous tdTomato signal and DAPI counterstain, shown in maximum intensity projections of 10 um confocal z-stacks, 0.5x zoom B) No tdTomato signal is observed in non-tamoxifen injected control male and female BCA counterstained with DAPI and Acta2-FITC and imaged as in A. C) Myh11-Dre^{ERT2} negative (-) Rosa-tdTomato-eGFP BCA and liver, counterstained with DAPI and Acta2-FITC show no labeling of cells with tdTomato in absence of Myh11-Dre^{ERT2}. D) Myh11-Dre^{ERT2} Lgals3-Cre Rosa-tdTomato-eGFP mice were injected with tamoxifen at 6-8 weeks of age and subsequently harvested for BCA, bladder, and multiple other SMC rich tissues such as uterus and small intestine. Fixed sections of BCA and bladder were stained for tdTomato and eGFP and imaged with 20x confocal microscopy and shown as maximum intensity projections of 10 um confocal z-stacks, 0.5x zoom. Myh11-Dre^{ERT2} Lgals3-Cre Rosa-tdTomato-eGFP ApoE$^{-/-}$ chow fed mice show efficient labeling of SMC rich tissues such as the bladder and aorta with tdTomato, with no stochastic GFP expression observed. E) The aorta from a Myh11-Dre^{ERT2} Lgals3-Cre Rosa-tdTomato-eGFP mouse was stripped of adventitia ex vivo and imaged with fluorescence microscopy in the FITC and Cy3 channels for endogenous tdTomato and eGFP respectively. F) DNA was isolated from aortae from Myh11-Dre^{ERT2} Lgals3-Cre Rosa-tdTomato-eGFP mice with and without tamoxifen injection and from Myh11-Dre^{ERT2} Rosa-tdTomato-eGFP mice without Lgals3-Cre. DNA was amplified with primers spanning the rox-STOP-rox cassette, generating a product of 800 bp unrecombined and 450 recombined. G) Lgals3-Cre Rosa-tdTomato-eGFP , Myh11-Dre^{ERT2} Rosa-tdTomato-eGFP , and Rosa-tdTomato-eGFP mice were injected with tamoxifen from 6-8 weeks of age and aortas were harvested for flow cytometry. No GFP^{+} cells are observed by flow cytometry of aortas from control mice missing one or both recombinases. H) Lgals3-Cre Rosa-tdTomato-eGFP ApoE$^{-/-}$ and Myh11-Dre^{ERT2} Rosa-tdTomato-eGFP ApoE$^{-/-}$ mice were injected with tamoxifen and fed a western diet for 18 weeks. Aorta was harvested for flow cytometry. No eGFP^{+} cells are visible in mice missing either recombinase I) Sections from BCA lesions from from 18 wk western diet fed Myh11-Dre^{ERT2} Rosa-tdTomato-eGFP ApoE$^{-/-}$ mice were immunostained for tdTomato and eGFP and counterstained with DAPI. Images are the maximum intensity projection image of the 10um tissue thickness at 0.5x zoom and 20x magnification. No eGFP^{+} cells are observed in atherosclerotic lesions without Lgals3-Cre.

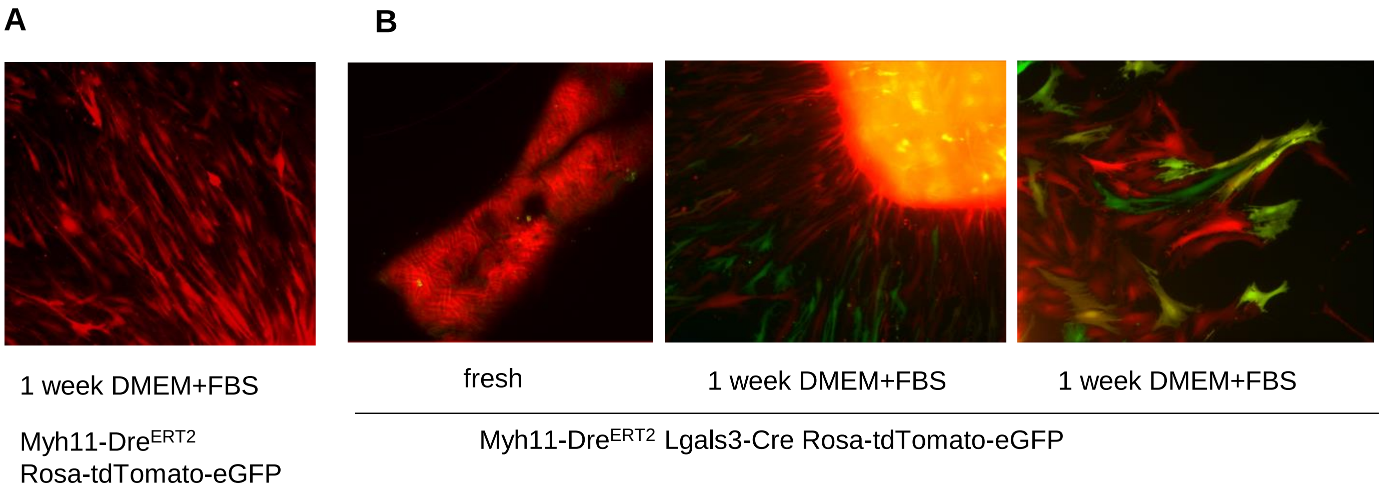

Supplemental Figure XIII. **Explants from Myh11-Dre^{ERT2} Lgals3-Cre Rosa-tdTomato-eGFP mice show tdTomato^{+}eGFP^{+} transition cells in culture**. A) Aortic explants were taken from a Myh11-Dre^{ERT2} Rosa-tdTomato-eGFP mouse, stripped of adventitia and placed in DMEM medium supplemented with 10% fetal bovine serum for 1 week. Myh11-Dre^{ERT2} Rosa-tdTomato-eGFP without Lgals3-Cre remain only tdTomato^{+}, even after a week of culture in 10% fetal bovine serum containing media. B) Aortic explants were taken from Myh11-Dre^{ERT2} Lgals3-Cre Rosa-tdTomato-eGFP mice, photographed as fresh tissue, and imaged after 1 week with fluorescent microscopy for endogenous tdTomato and eGFP signal. Explants from Myh11-Dre^{ERT2} Lgals3-Cre Rosa-tdTomato-eGFP mice show tdTomato^{+}eGFP^{+} cells (yellow).

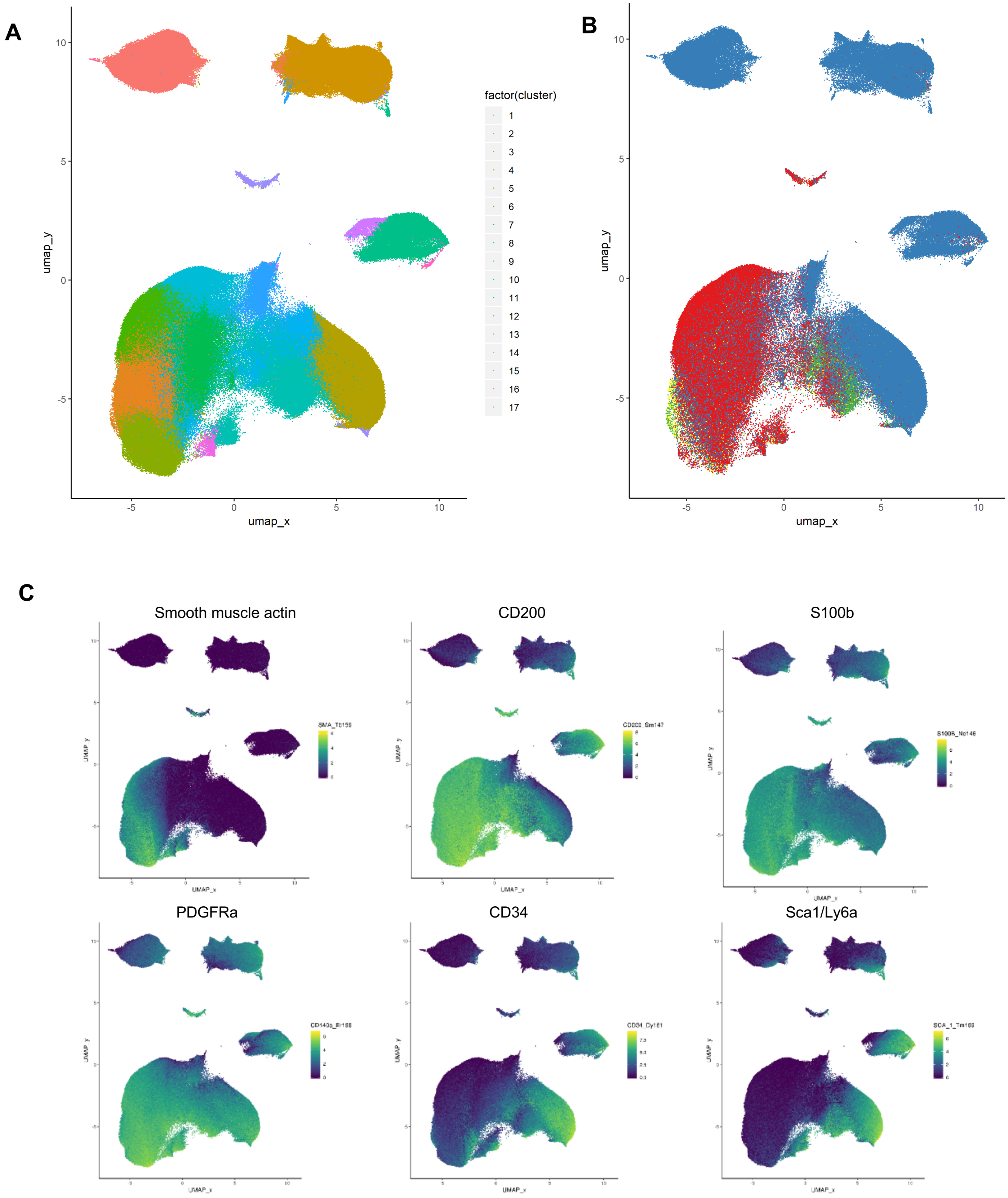

Supplemental Figure XIV. **Expression of selected CyTOF markers in Myh11-DreERT2 Lgals3-Cre Rosa-tdTomato-eGFP ApoE$^{-/-}$ atherosclerotic aortas** A) Aortas from 18-week western diet fed Myh11-DreERT2 Lgals3-Cre Rosa-tdTomato-eGFP ApoE$^{-/-}$ mice were analyzed with the same CyTOF panel as in Supplemental Figure 8, using antibodies to RFP and GFP to detect tdTomato and eGFP, respectively. Results are shown as UMAP of all aortic cells, colored by UMAP cluster. B) Expression of tdTomato(red), GFP(green), both (yellow), or neither(blue). C) Expression of selected markers by CyTOF is shown on a UMAP colored by level of expression (yellow = higher expression level).

A

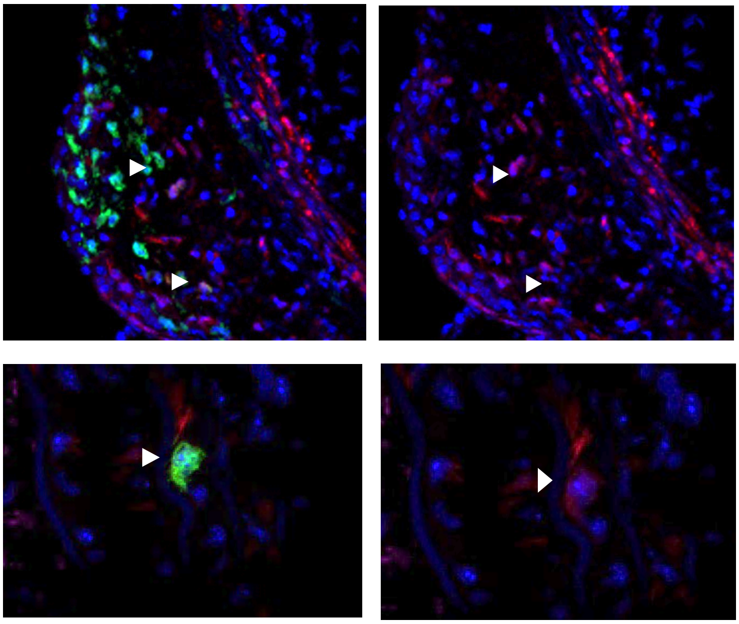

B

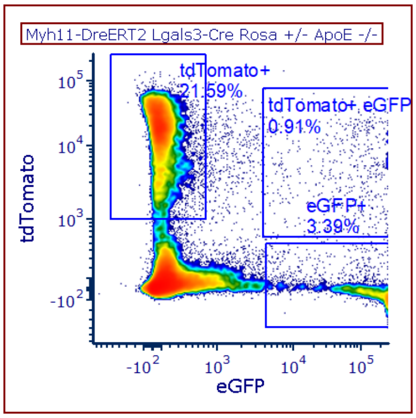

Supplemental Figure XV. **Double positive tdTomato$^+$ eGFP$^+$ cells are visible even in mice heterozygous at the Rosa-tdTomato-eGFP locus, and are not cells recombined at only one allele.** A) Sections from BCA lesions from 18 wk western diet fed Myh11-Dre^{ERT2} Lgals3-Cre Rosa-tdTomato-eGFP$^{+/-}$ ApoE$^{-/-}$ mice heterozygous at the Rosa locus were immunostained for tdTomato and eGFP, counterstained with DAPI, and imaged in 1 µm confocal z-stacks of full tissue thickness at 20x, 0.5x zoom. Single z stacks are shown in close up image, max intensity projections for the full lesion picture. B) Flow cytometry of microdissected BCA lesions from Myh11-Dre^{ERT2} Lgals3-Cre Rosa-tdTomato-eGFP$^{+/-}$ ApoE$^{-/-}$ mice heterozygous at the Rosa locus. eGFP and tdTomato were detected as endogenous signal in the FITC and Cy3 channels.

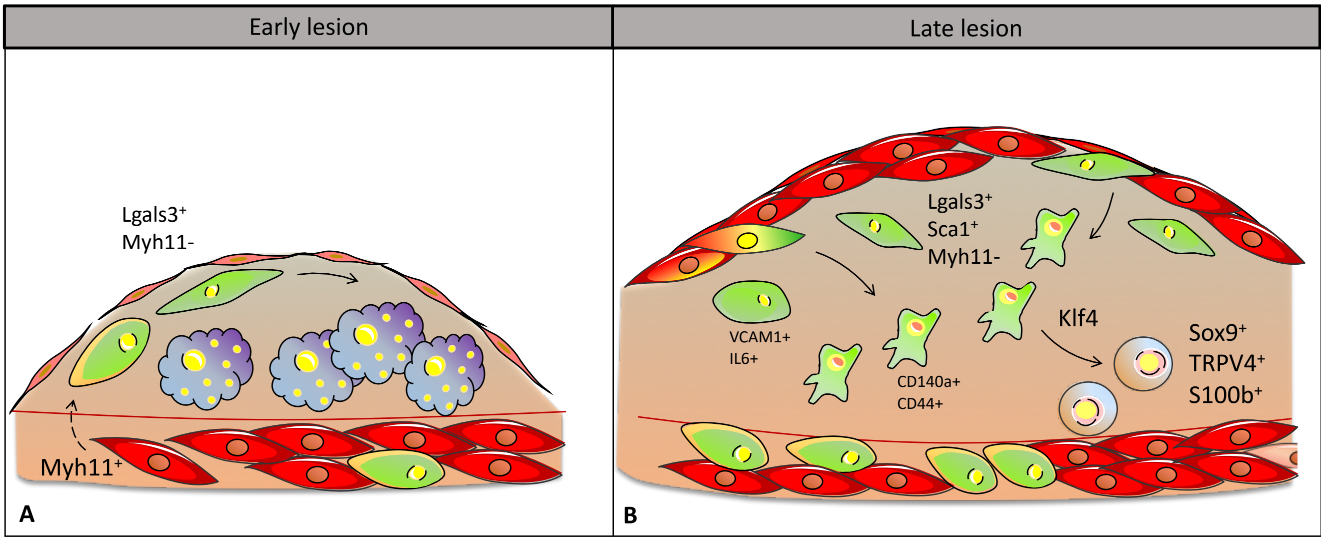

Supplemental Figure XVI. **Summary of SMC phenotypic switching through Lgals3 in atherosclerosis.** A) In early atherogenesis, Lgals3-switched cells invade the subendothelial space and recruit SMC into the lesion. B) In late stage disease, Myh11$^+$ SMC make up the majority of the fibrous cap while Lgals3$^+$ switched cells take on an ECM-rich, osteochondrogenic state dependent on Klf4. Myh11$^+$ cap cells may also undergo transition within the lesion to an Lgals3$^+$ state.

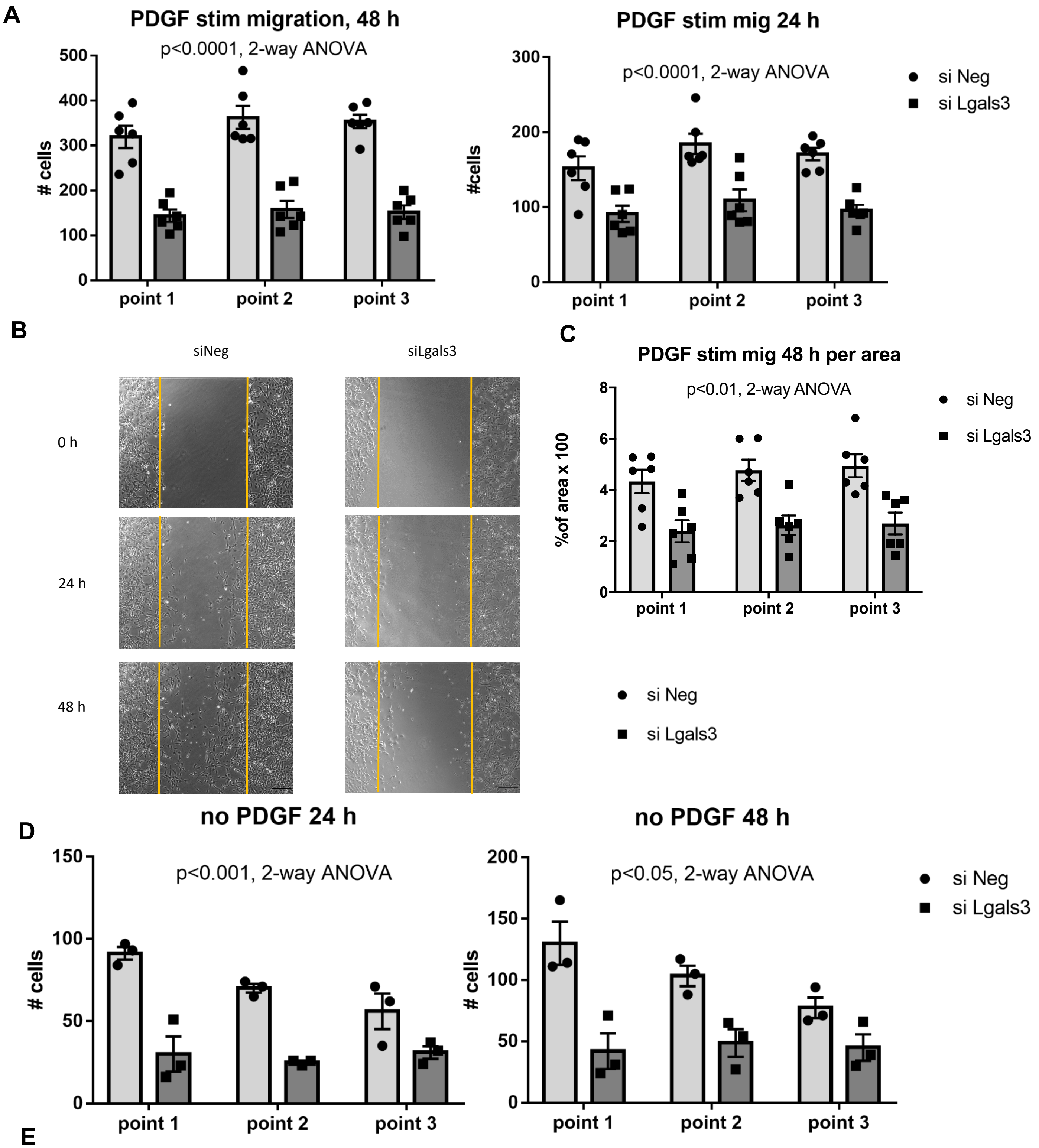

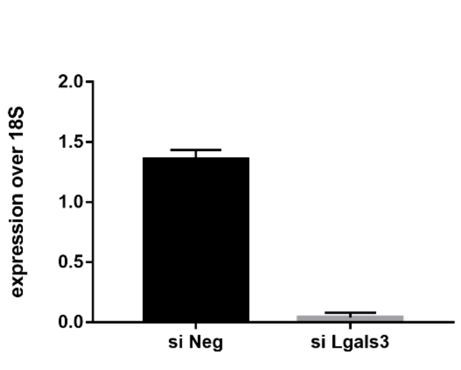

Supplemental Figure XVII. **siRNA-induced knockdown of Lgals3 in cultured mouse aortic SMC significantly decreased scratch wound healing.** A)SMC isolated from aorta from our SMC lineage tracing mice were treated with siLgals3 or siNeg scramble control, serum starved for 24 hours, scratched with a pipet tip and treated with 10 ug/ml PDGF-BB in DF10 media with 10% serum. Light microscopy images were taken at three different points along the scratch, each at time 0, 24, and 48 hours. Results were quantified as the number of cells that had entered the scratch area as defined at time 0 hours, n=6. B) Representative images of siNeg and siLgals3 scratch C) Results in A normalized for total scratch wound area. D) Results were similar if SMC lineage traced cells were scratched as in A but not treated with PDGF-BB. P values for A-D refer to differences between experimental groups (siNeg vs siLgals3). E) siLgals3 and siNeg treated SMC were analyzed by qRT-PCR for Lgals3 (normalized to a housekeeping gene 18s).

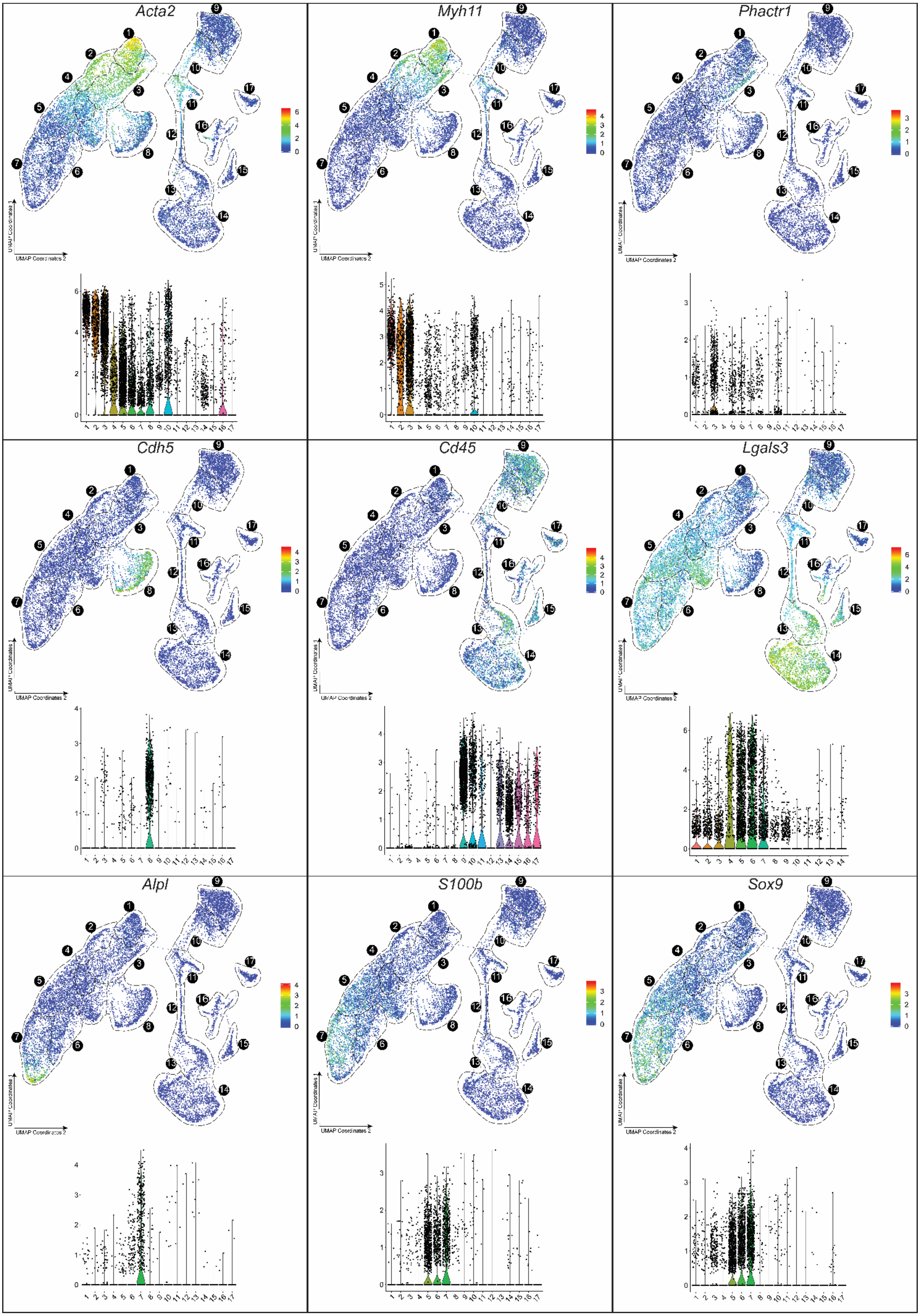

Supplemental Figure XVIII. **UMAP showing traditional marker genes for SMC, endothelial, and macrophages with all mouse and human cells, as well as markers shown in Figures 3, 5-6.** Top row shows the expression of *Acta2*, *Myh11*, and *Phactr1*. These genes are enriched in clusters 1-3. Middle row shows the expression of *Cdh5* (endothelial marker), *Cd45* (macrophage marker), and *Lgals3*. Bottom row shows the expression of *Alpl, S100b, and Sox9*. These genes are enriched in the cluster 6-7 and are associated with calcification.
